# A Covalent and Modulable Inhibitor of the Tubulin‐Microtubule System: Insights Into the Mechanism of Cacalol

**DOI:** 10.1111/cbdd.70165

**Published:** 2025-09-11

**Authors:** Edgar López‐López, José L. Medina‐Franco, Eric Salinas‐Arellano, Karen J. Ardila‐Fierro, Julio C. Pardo‐Novoa, Rosa E. del Río, Carlos M. Cerda‐García‐Rojas

**Affiliations:** ^1^ Departamento de Química y Programa de Posgrado en Farmacología Centro de Investigación y de Estudios Avanzados del Instituto Politécnico Nacional Mexico City Mexico; ^2^ DIFACQUIM Research Group, Department of Pharmacy, School of Chemistry Universidad Nacional Autónoma de México Mexico City Mexico; ^3^ Departamento de Ciencias de la Tierra y de la Vida Centro Universitario de los Lagos, Universidad de Guadalajara Lagos de Moreno Jalisco Mexico; ^4^ Grupo Ciencia de los Materiales, Instituto de Química, Facultad de Ciencias Exactas y Naturales Universidad de Antioquia Medellín Colombia; ^5^ Instituto de Investigaciones Químico‐Biológicas Universidad Michoacana de San Nicolás de Hidalgo, Ciudad Universitaria Morelia Michoacán Mexico

**Keywords:** covalent inhibitors, molecular dynamics, naphthofuran derivatives, peptide mass spectrometry, polymerization inhibitors, ROS‐dependent inhibitors, tubulin‐microtubules system

## Abstract

Inhibitors of the tubulin‐microtubule system are part of an effective strategy to treat different kinds of cancer, whose research has allowed scientists to discover and develop new and more selective molecules. Cacalol (**1**) is a natural product with anti‐cancer activity and documented selectivity in breast cells, but with an undescribed molecular mechanism associated with these properties. The main objective of this work is to provide evidence that helps to explain the inhibitory and selective activity reported for cacalol (**1**) against cancer cell lines and to expand the knowledge about the mechanism of action involved in it. Cacalol derivatives were studied using reactivity approaches, tubulin polymerization assays, mass spectrometry, and molecular modeling techniques to decode the inhibitory binding mechanism. This work demonstrates that an oxidated form of cacalol, the methylenecyclohexadienone **2**, is generated in highly oxidant conditions, thus emulating the environment present in cancer cells. This species (**2**) is responsible for the inhibition of tubulin polymerization by promoting an irreversible binding interaction with the Cys347 in α‐tubulin.

## Graphical abstract

An oxidated form of cacalol, the methylenecyclohexadienone MTC (**2**), is generated under highly oxidant conditions, thus emulating the environment present in cancer cells. This species (**2**) is responsible for the inhibition of tubulin polymerization by promoting an irreversible binding interaction with Cys347 in α‐tubulin.

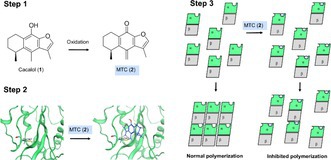

## 1 Introduction

**FIGURE 1:**
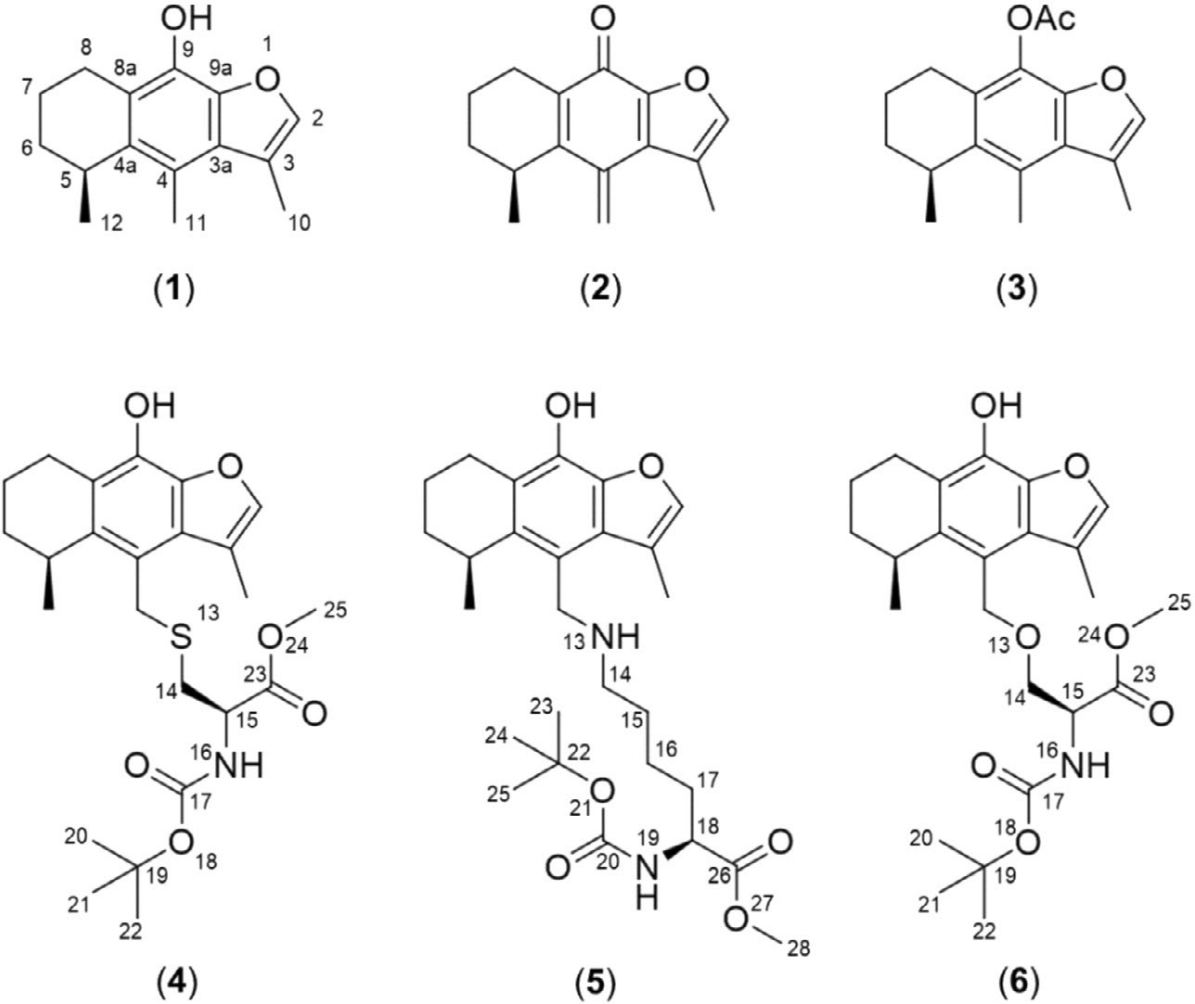
Chemical structures of cacalol (**1**); methylenecyclohexadienone derivative (MTC) (**2**); cacalol acetate (**3**); MTC–cysteine derivative adduct (**4**); MTC–lysine derivative adduct (**5**); MTC–serine derivative adduct (**6**).

Cacalol (**1**) and its chemical derivatives (Figure 1) have been associated with multiple biological applications, for example, anti‐inflammatory (Alarcon‐Aguilar et al. 2010; Jimenez‐Estrada et al. 2006; Mora‐Ramiro et al. 2020), antioxidant (Shindo et al. 2004), antifungal (Anaya et al. 1996), anti‐cancer (Liu et al. 2010; Rostro‐Alonso et al. 2024), antihyperglycemic (Inman et al. 1999), and anti‐allergic (Castillo‐Arellano et al. 2018) activities. These compounds inhibit the expression of proinflammatory cytokines, inhibit lipid peroxidation, and promote the re‐establishment of energy metabolism. However, their biological endpoints have not been clarified yet.

Tubulin is a structural protein with different isoforms, mainly the α and β units, that polymerize to form microtubules involved in cell division, apoptosis, indirect regulation of energy metabolism, and immune system signaling and regulation (Janke and Magiera 2020). On the other hand, reactive oxygen species (ROS) are a cornerstone to understand chronic degenerative diseases such as cancer. In fact, there is a plethora of reports that demonstrate the therapeutic potential of antioxidant agents to treat these kinds of illnesses (Ranneh et al. 2017). Interestingly, cacalol (**1**) is an antioxidant compound that inhibits the generation of free radicals (Gómez‐Vidales et al. 2014). Also, the properties displayed by cacalol (**1**) suggest that the reported biological effects of this substance could be conducted by interaction with the tubulin‐microtubule (Tub‐Mts) system through a mechanism involving oxidant species (Liu et al. 2010).

In the present work, we explore the mechanism of action of cacalol (**1**) and two derivatives (**2** and **3**) by using tubulin polymerization assays, reactivity approaches, mass spectrometry, and molecular dynamics to describe their covalent binding interaction with α‐tubulin. These results confirm that cacalol (**1**) bioactivity is explained by a covalent binding mechanism of an oxidated form of cacalol, namely the methylenecyclohexadienone **2**, generated by the presence of oxidant species.

## 2 Materials and Methods

### 2.1 Experimental Procedures

#### 2.1.1 General Experimental Procedures

NMR spectra were measured at 300 MHz for ^1^H and 75.4 Hz for ^13^C on a Varian Mercury 300 spectrometer or at 500 MHz for ^1^H and 125 MHz for ^13^C on a Jeol ECA 500 spectrometer from CDCl_3_ solutions containing tetramethylsilane as the internal reference. Chemical shift values are reported in ppm and coupling constants (*J*) are in Hz. HRESIMS spectra were measured on an Orbitrap Exploris 120 mass spectrometer. Optical rotations were recorded in CHCl_3_ solutions on a PerkinElmer 341 polarimeter. IR spectra were obtained on a BioTools dualPEM Chiral*IR* FT spectrophotometer in CHCl_3_ solutions. UV spectra were measured in EtOH on a PerkinElmer Lambda 12 spectrophotometer. Silica gel 230–400 mesh (Merck) was used for column chromatography.

#### 2.1.2 Cacalol (1)

^1^H NMR and ^13^C NMR data of cacalol (**1**) were in agreement with literature values (Liu et al. 2010; del Río et al. 2021; Omura et al. 1978; Yuste et al. 1976); see Figures S1–S3 in the Supporting Information section.

#### 2.1.3 Methylenecyclohexadienone Derivative (MTC) (2) or (*S*)‐3,5‐Dimethyl‐4‐Methylene‐5,6,7,8‐Tetrahydronaphtho[2,3‐b]Furan‐9(4*H* )‐One (2)

A solution of **1** (20 mg, 0.087 mmol) in CH_2_Cl_2_ (2 mL) was stirred in the presence of finely ground Ag_2_O (80 mg; 0.345 mmol) at room temperature for 3 h. The reaction mixture was filtered; the filtrate was evaporated and applied to a silica gel column eluting with hexanes‐EtOAc (92:8). Fractions 9–23 yielded **2** as a colorless oil (18 mg, 0.079 mmol; 90%); ^1^H NMR: δ ppm 7.41 (q, *J* = 1.2 Hz, 1H, H‐2), 6.28 (br s, 1H, H‐11a), 6.19 (br s, 1H, H‐11b), 3.15 (m, 1H, H‐5), 2.79 (br dd, 1H, *J* = 21.2, 3.8 Hz, H‐8a), 2.38 (m, 1H, H‐8b), 2.29 (d, *J* = 1.2 Hz, 3H, Me‐10), 1.83–1.71 (m, 4H, H‐6a, H‐6b, H‐7a, and H‐7b), 1.27 (d, *J* = 7.0 Hz, 3H, Me‐12); ^13^C NMR δ ppm 175.5 (CO, C‐9) 147.4 (C, C‐9a), 146.6 (C, C‐4a), 144.2 (CH_2_, C2), 135.2 (C, C‐4), 134.3 (C, C‐8a), 129.6 (C, C‐3a), 122.0 (CH_2_, C‐11), 118.9 (C, C‐3), 29.3 (CH_2_, C‐6), 27.9 (CH_2_, C‐5), 23.0 (CH_2_, C‐8), 23.0 (CH_3_, C‐12), 16.4 (CH_2_, C‐7), 10.9 (CH_3_, C‐10); IR (CHCl_3_) ν_max_ 2932 (CH), 1658 and 1637 (C=O), 1461 (C=CH), 1233, 1220, 1202, and1118 cm^−1^ (C–O); [α]_589_ = +21, [α]_578_ = +22, [α]_546_ = +28 (*c* 0.12, EtOH); UV λ_max_ (EtOH) 221 (log ε 3.85), 299 nm (log ε 3.73). HRESIMS *m/z* 229.1232 [M + H]^+^ (calcd for C_15_H_16_O_2_ + H^+^, 229.1228); see Figures S4–S8 in the Supporting Information section.

#### 2.1.4 Cacalol Acetate (3)

^1^H NMR and ^13^C NMR data were in agreement with literature values (Liu et al. 2010; del Río et al. 2021; Omura et al. 1978; Yuste et al. 1976); see Figures S9–S11 in the Supporting Information section.

### 2.2 Covalent Bonding Between 2 and Protected Amino Acid Derivatives

Compound **2** (MTC) was allowed to react with L‐cysteine (Cys), L‐lysine (Lys), or L‐serine (Ser) protected derivatives to generate compounds **4**, **5**, or **6**, respectively (Figure 1).

#### 2.2.1 Compound 4

A solution of **2** (10 mg, 0.044 mmol) in CH_2_Cl_2_ (1 mL) was treated with *N*‐Boc‐Cys‐OMe (10 mg, 0.908 mmol) at room temperature for 3 h. The solvent was evaporated, and the residue was chromatographed on a silica gel column eluting with mixtures of hexanes–EtOAc. Compound **4** was obtained in fractions 8–11 eluted with hexanes–EtOAc (85:15) as a yellow oil (10 mg, 0.021 mmol, 49%): ^1^H NMR: δ ppm 7.27 (q, *J* = 1.4 Hz, 1H, H‐2), 5.37 (d, *J* = 7.7 Hz, 1H, NH), 5.18 (br s, 1H, OH), 4.58 (dt, *J* = 7.7, 5.1 Hz, 1H, H‐15), 4.19 (d, *J* = 11.4 Hz, 1H, 11a), 4.02 (d, *J* = 11.4 Hz, 1H, 11b), 3.75 (s, 3H, OMe), 3.37 (m, 1H, H‐5), 3.12 (dd, *J* = 14.2, 4.9 Hz, 1H, H‐14a), 3.03 (dd, *J* = 14.2, 5.3 Hz, 1H, H‐14b), 2.95 (br ddd, *J* = 17.5, 6.0, 1.7 Hz, 1H, H‐8a), 2.65 (ddd, *J =* 17.5, 10.5, 7.5 Hz,1H, H‐8b), 2.48 (d, *J* = 1.3 Hz, 3H, Me‐10), 1.98–1.74 (m, 4H, H‐6a, H‐6b, H‐7a, H‐7b), 1.46 (s, 9H, Me‐20, Me‐21, Me‐22), 1.27 (d, *J* = 6.9 Hz, 3H, Me‐12); ^13^C NMR δ ppm 171.7 (C=O, C‐23), 155.2 (C=O, 17), 142.5 (C, 9a), 141.5 (CH, 2), 138.1 (C, 9), 137.2 (C, 4a), 126.3 (C, 3a), 119.0 (C, 4), 118.3 (C, 8a), 116.6 (C, 3), 80.3 (C, 19), 53.6 (C, 15), 52.7 (C, 25), 35.5 (CH_2_, 14), 30.6 (CH_2_, 11), 29.6 (CH_2_, 5), 29.3 (CH_2_, 6), 28.4 (CH_3_, 20, 21, 22), 22.9 (CH_2_, 12), 22.5 (CH_3_, 8), 16.3 (CH_3_, 7), and 10.2 (CH_3_, 10). IR (CHCl_3_) ν_max_ 3578 (OH), 3440 (NH), 2934 (CH_2_ and CH_3_), 1744 (COO), 1711 (NCOO), 1499 (N‐H), 1234 (C‐O), 1165 (C‐O) cm^−1^; [α]_589_ + 6.4, [α]_578_ + 6.7, [α]_546_ + 8.1, [α]_436_ + 19.1 (*c* 0.13, CHCl_3_); UV (EtOH) λ_max_ (log ε) 223 (4.36) nm, 268 (3.96) nm; HRESIMS *m/z* 480.2014 [M(O) + H]^+^ (calcd for C_24_H_33_NO_7_S + Na, 480.2050). See Figures S12–S15 in the Supporting Information section. The oxidation of cysteine derivatives with a sulfide moiety during HRESIMS methodologies was previously documented, being in agreement with our data (Hee‐Jung et al. 2015).

#### 2.2.2 Compound 5

A solution of **2** (10 mg, 0.044 mmol) and *N*‐Boc‐Lys‐OMe•HCl (30 mg, 0.101 mmol) in CH_2_Cl_2_ (1 mL) was stirred at room temperature for 24 h. The reaction mixture was processed as for compound **4**. The product was purified by column chromatography eluting with EtOAc. Fractions 5–8 yielded pure **5** as an oil (2 mg, 0.004 mmol, 9%) ^1^H NMR: δ ppm 7.18 (br s, 1H, H‐2), 5.03 (br d, *J* = 8.1 Hz, 1H, NH), 4.29 (m, 1H, H‐18), 3.97 (br s, 2H, H‐11a, H‐11b), 3.72 (s, 3H, OMe), 3.31 (m, 1H, H‐5), 2.92 (br dd, *J* = 17.5, 5.4 Hz, 1H, H‐8a), 2.78 (t, *J* = 6.9 Hz, 2H, H‐14), 2.57 (ddd, *J* = 17.5, 10.2, 8.1 Hz,1H, H‐8b), 2.37 (d, *J* = 1.2 Hz, 3H, Me‐10), 1.90–1.74 (m, 4H, H‐6a, H‐6b, H‐7a, H7‐b), 1.73–1.52 (m, 6H, H‐15a, H‐15b, H‐16a, H‐16b, H‐17a, H‐17b), 1.43 (s, 9H, Me‐23, Me‐24, Me‐25), 1.23 (d, *J* = 6.9 Hz, 3H, Me‐12); ^13^C NMR: 173.6 (C, 26), 155.5 (C, 20), 144.1 (CH, 9a), 141.8 (C, 2), 141.2 (C, 3), 138.3 (C, 4), 136.6 (C, 4a), 126.2 (C, 8a), 121.8 (C, 22), 119.3 (C, 9), 115.8 (C, 3a), 53.3 (CH, 18), 52.2 (CH_2_, 17), 49.2 (CH_2_, 16), 45.5 (CH_2_, 15), 32.5 (CH_2_, 14), 31.9 (CH_2_, 11), 29.7 (CH_2_, 5), 28.5 (CH_2_, 8), 28.3 (CH_3_, 23–25), 23.4 (CH_3_, 6), 23.0 (CH_3_, 12), 22.9 (CH_2_,7),16.4 (CH_3_, 10), 10.3 (CH_3_, 28); IR (CHCl_3_) ν_max_ 3542 (OH), 3435 (NH), 2932 (CH_2_ and CH_3_), 1747 (COO), 1712 (NCOO), 1454 (N‐H), 1234 (C‐O), 1165 (C‐O), 1116 (C‐O) cm^−1^; [α]_589_ + 10.0, [α]_578_ + 11.1, [α]_546_ + 12.2 (*c* 0.09, CHCl_3_); UV (EtOH) λ_max_ (log ε) 220 (5.35) nm, 262 (4.88) nm, 299 (4.62) nm; HRESIMS *m/z* 489.2918 [M + H]^+^ (calcd for C_27_H_40_N_2_O_6_ + H^+^, 489.2959). See Figures S16–S19 in the Supporting Information section.

#### 2.2.3 Compound 6

A solution of **2** (20 mg, 0.088 mmol) and *N*‐Boc‐Ser‐OMe (50 mg, 0.228 mmol) in CH_2_Cl_2_ (1 mL) was stirred at room temperature for 150 h. The reaction mixture was processed as for compound **4**. The product was purified by column chromatography eluting with hexanes‐EtOAc (85:15). Fractions 6–11 yielded **6** as an oil (2 mg, 0.004 mmol, 5%): ^1^H NMR: *δ* ppm 7.28 (br s, 1H, H‐2), 5.35 (d, *J* = 8.7 Hz, 1H, NH), 5.23 (s, 1H, OH), 4.74 (s, 2H, H‐11a, H‐11b), 4.42 (dt, *J* = 8.7, 3.3 Hz, 1H, H‐15), 3.90 (dd, *J* = 9.3, 3.3 Hz, 1H, H‐14a), 3.74 (dd, *J* = 9.3, 3.3 Hz, 1H, H‐14b), 3.61 (s, 3H, 25), 3.35 (m, 1H, H‐5), 2.97 (ddd, *J* = 17.5, 6.0, 2.7 Hz, 1H, H‐8a), 2.65 (ddd, *J* = 17.5, 10.8, 7.8 Hz, 1H, H‐8b), 2.29 (d, *J* = 1.5 Hz, 3H, Me‐10), 1.96–1.76 (m, 4H, 6a, 6b, 7a, 7b), 1.42 (s, 9H, Me‐20, 21, 22), and 1.22 (d, *J* = 6.9 Hz, 3H, Me‐12); ^13^C NMR δ ppm 171.1 (C=O, 23), 155.5 (C=O, 17), 142.3 (C, 9a), 141.4 (CH, 2), 138.6 (C, 9), 137.8 (C, 4a), 127.3 (C, 3a), 118.9 (C, 8a), 118.6 (C, 4), 116.8 (C, 3), 79.9 (C, 19), 69.3 (CH_2_, 11), 65.6 (CH_2_, 14), 54.1 (CH, 15), 52.2 (CH_3_, 25), 29.8 (CH_2_, 6), 28.6 (CH, 5), 28.3 (CH_3_, 20, 21, 22), 23.4 (CH_2_, 12), 22.7 (CH_3_, 8), 16.4 (CH_2_, 7), and 9.8 (CH_3_, 10); IR (CHCl_3_) ν_max_ 3435 (NH, OH), 2933 (CH_2_ and CH_3_), 1747 (COO), 1714 (NCOO), 1160 (C‐O) cm^−1^; [α]_589_ + 67.4, [α]_578_ + 71.4, [α]_546_ + 86.3, [α]_436_ + 222.9 (*c* 0.35, CHCl_3_); UV (EtOH) λ_max_ (log ε) 214 (6.75) nm; HRESIMS *m/z* 470.2114 [M + Na]^+^ (calcd for C_24_H_33_NO_7_ + Na^+^, 470.2149) See Figures S20–S23 in the Supporting Information section.

### 2.3 Competitive Covalent Reaction With Protected Amino Acid Derivatives

A solution of **2** in CH_2_Cl_2_ (3 mL) was stirred at room temperature for 5 h in the presence of *N*‐Boc‐Cys‐OMe, *N*‐Boc‐Lys‐OMe·HCl, and *N*‐Boc‐Ser‐OMe in equimolar concentrations with respect to **2**. The reaction product was purified by column chromatography according to the protocol previously mentioned (*vide supra*) to yield **4** as the only product.

### 2.4 Cacalol (1) Oxidation Using 2,2‐Diphenyl‐1‐Picrylhydrazyl Radical

A solution of **1** in CH_2_Cl_2_ was treated with the 2,2‐diphenyl‐1‐picrylhydrazyl radical (DPPH; CAS number: 1898‐66‐4) in a 1:3 M ratio at room temperature during 24 h. The identity of the product (**2**) was verified using ^1^H NMR and by comparison with a pure sample obtained as described in Section 2.1.3.

### 2.5 Purity of Compounds Subjected to Tubulin Polymerization Evaluations

Purity analysis of cacalol (**1**) was carried out with an UHPLC UltiMate 3000 Thermo Scientific instrument with a diode array detector using a ZORBAX XDB‐C18 column (3.5 μm, 1.2 × 100 mm) eluting with HPLC grade acetonitrile/water/formic acid (v/v/v, 55:45:0.1%) at a flow rate of 0.6 mL/min, column temperature 35°C, detection wavelength at 221 nm, and an injection volume of 20 μL. The purity of cacalol (**1**) was 97.8% (Rt = 3.27 min) as seen in Figure S3 (Supporting Information section). Purity analyses of MTC (**2**) and cacalol acetate (**3**) were performed with an HPLC Agilent 1200 series and quadrupole LC/MS 6120 equipment using a ZORBAX XDB‐C18 column (5 μm, 4.6 × 150 mm) eluting with HPLC grade acetonitrile/water/formic acid (v/v/v, 70:30:0.05%) at a flow rate of 0.4 mL/min, and an injection volume of 0.20 μL. The purity of MTC (**2**) was estimated to be 100% (Rt = 11.58) as shown in Figure S8, while that for cacalol acetate (**3**) was 96.1% (Rt = 15.07 min) as seen in Figure S11 (Supporting Information section).

### 2.6 Tubulin Polymerization Assays

The purity stock solutions (11 mM) of cacalol (**1**) and derivatives **2** and **3** in DMSO (for molecular biology) were separately prepared. Then, 10 μL of each solution were placed on 96‐well half‐area ELISA plates to be combined with the tubulin solutions. The experiments used porcine brain α, β‐tubulin 97% (Cytoskeleton Inc.) as indicated in a described protocol (Silva‐García et al. 2019). Freshly reconstituted solutions of the protein in a tubulin general buffer at pH 6.9 were added in aliquots of 100 μL at 0°C to the wells to give a final concentration of 100 μM of each tested compound and a final tubulin concentration of 2 mg/mL. Additionally, the most active compound (**2**) was studied at five final concentrations (10, 25, 50, 100, and 200 μM, respectively). The tubulin general buffer consisted of 80.0 mM piperazine‐*N*,*N*′‐bis(2‐ethanesulfonic acid) sesquisodium salt, 2.0 mM MgCl_2_, 0.5 mM ethylene glycol‐bis(β‐aminoethyl ether)‐*N*,*N*,*N*′,*N*′‐tetraacetic acid, and 1.0 mM guanosine 5′‐triphosphate. The plates were analyzed using a BioTek EL808 IU microplate reader preheated at 37°C. Tubulin polymerization was monitored by measuring the absorbance at 450 nm every minute for 60 min. DMSO (10 μL) was used as the negative control, nocodazole (10 μM) as the anti‐polymerization control, and paclitaxel (10 μM) as the polymerization control. The reading at time zero was subtracted from subsequent readings to obtain Δ absorbance. All assays were carried out in triplicate, and graphs were prepared in GraphPad Prism software v.9. The results were evaluated using an analysis of variance followed by a Tukey's test, *p* < 0.001.

### 2.7 Mass Spectrometry

Purified porcine tubulin (Cytoskeleton Inc.) was dissolved into a general tubulin buffer without its co‐factor (GTP) to obtain a final concentration of 3 mg/mL. The solution was incubated at room temperature for 3 h in the presence of MTC (**2**) in DMSO (10 μL) to keep a molar ratio of 1:1 and 1:100 of tubulin:MTC. An equivalent sample of the protein solution was treated only with DMSO as the negative control. After that, the unreacted MTC (**2**) was quenched by free cysteine. After the reaction and quenching, the protein for each condition was purified by SDS‐PAGE. Gel slices from the ~55 kDa migration position were enzymatically digested according to a modified protocol (Winkler et al. 2007). Afterwards, the digested peptides were loaded into a Symmetry C18 Trap V/M precolumn (Waters, Milford, MA); 180 μm × 20 mm, 100 Å pore size, 5‐μm particle size desalted using as a mobile phase A 0.1% formic acid in H_2_O (v/v) and mobile phase B and 0.1% formic acid in acetonitrile under the following isocratic gradient: 99.9% mobile phase A and 0.1% mobile phase B at a flow rate of 5 μL/min for 3 min. Subsequently, peptides were loaded and separated on an HSS T3 C18 Column (Waters, Milford, MA), 75 μm × 150 mm, 100 Å pore size, 1.8‐μm particle size, using a UPLC ACQUITY M‐Class (Waters, Milford, MA) with the similar mobile phases under the following gradient: 0 min 7% B, 30 min 40% B, 32–35 min 85% B, 37–47 min 7% B at a flow rate of 400 nL/min and 45°C. Spectral data were acquired using a Synapt G2‐Si (Waters, Milford, MA) mass spectrometer with electrospray ionization and ion mobility separation using a data‐independent acquisition approach in the high‐definition MS mode. The tune page for the ionization source was set up with the following parameters: 2.75 kV in the sampler capillary emitter, 30 V in the sampling cone, 30 V in the source offset, 70°C for the source temperature, 0.5 bar for the nanoflow gas, and 120 L/h for the purge gas flow rate.

Two chromatograms were acquired (low‐ and high‐energy chromatograms) in positive mode in the 50–2000 *m/z* range with a scan time of 500 ms. No collision energy was applied to obtain the low‐energy chromatogram. However, in the case of the high‐energy chromatograms, the precursor ions were fragmented in the transfer cell using a collision energy ramp of 19–55 V. Generated *.raw files containing MS and tandem mass spectra were deconvoluted and compared using ProteinLynx Global SERVER v3.0.3 (Waters, Milford, MA) against a reversed *Sus scrofa* UP000008227 *.fasta database (downloaded from UniProt 46,179 protein sequences) concatenated with its reversed database. Workflow parameters were trypsin as a cut enzyme, and one missed cleavage allowed. Automatic peptide and fragment tolerance, minimum fragment ion matches per peptide 2, minimum fragment ion matches per protein 5, minimum peptide matches per protein 1, and a false discovery rate ≤ 4%. All identifications had a ≥ 95% reliability (Protein AutoCurate green). Synapt G2‐Si was calibrated using [Glu1]‐fibrinopeptide, [M + 2H]^2+^ = 785.84261 at 1.3 ppm.

### 2.8 Molecular Modeling

#### 2.8.1 Molecular Docking

The crystallographic structure of a microtubule (PDB ID: 6O2S) was obtained from the Protein Data Bank database (Eshun‐Wilson et al. 2019). Water molecules and ligands were removed, while the hydrogen atoms and atom charges were assigned using the AMBER14 forcefield implemented in the Yasara software (Krieger and Vriend 2014). The molecular model of MTC (**2**) was constructed from its SMILES code and minimized using the MMFF94X force field. The molecular docking was generated using default parameters implemented in the AutoDock Vina protocol. The residues around the Cα of the Cys347 in the α‐tubulin (15 × 15 × 15 Å) were explored. For each ligand, 25 induced fit conformational searches were generated; the conformations with better binding scores were manually analyzed.

#### 2.8.2 Covalent Interaction Modeling

According to the mass spectrometry results, and the in vitro reactivity knowledge (*vide infra*), compound **2** was bonded to the sulfur atom of the Cys347 using the module “builder” implemented in the MOE software (Chemical Computing Group Inc. 2023). This complex was transformed into PDB format using the Open Babel v. 2.3.1 software (O'Boyle et al. 2011), and the final file was used for prospective molecular dynamics (MD) studies.

#### 2.8.3 Molecular Dynamics

MD studies of the protein‐ligand complexes were performed using Desmond (version 2021‐1, Schrödinger, New York, NY, USA) with the OPLS 2005 forcefield (López‐López et al. 2023). The complex was prepared using the “system builder utility”, buffering it with an orthotopic box (10 × 10 × 10 Å) using the transferable intermolecular potential with a 3‐point (TIP3P) model for water. The complexes were neutralized and NaCl was added in a 0.15 M concentration. Complexes were minimized using the steep‐descent (SD) algorithm followed by the Broyden‐Fletcher‐Goldfarb‐Shanno (L‐BFGS) method in three stages. In the first stage, water‐heavy atoms were restrained with a force constant of 1000 kcal mol^−1^ Å^−2^ for 5000 steps (1000 SD, 4000 L‐BFGS) with a convergence criterion of 50 kcal mol^−1^ Å^−2^. For the second stage, backbones were constrained with a 10 kcal mol^−1^ Å^−2^ force constant using a convergence criterion of 10 kcal mol^−1^ Å^−2^ for 2000 steps (1000 SD, 1000 L‐BFGS); and for the third stage, the systems were minimized with no restraints for 1000 steps (750 SD, 250 L‐BFGS) with a convergence criterion of 1 kcal mol^−1^ Å^−2^. Equilibration was carried out in several steps, beginning with Brownian Dynamics for 250 ps with the Berendsen thermostat, followed by simulation on the NVT ensemble, slowly heating from 10 to 300 K over 3000 ps. At this stage, constraints were enforced on solute‐heavy atoms, using a constant of 50 kcal/mol. Finally, equilibration on the NPT ensemble used the Berendsen thermostat and Langevin barostat for an additional 250 ps. Subsequently, the system was submitted to 150 ns of production runs under NPT ensemble at 1 bar using the Martyna‐Tuckerman‐Klein (MTK) barostat and 300 K using the Nosé‐Hoover thermostat. Electrostatic forces were calculated with the smooth PME method using a 9 Å cut‐off, while constraints were enforced with the M‐SHAKE algorithm. Integration was done every 1.2 fs, with a recording interval of 50 ps. The quality of simulations was achieved with the tools implemented in the Maestro‐GUI (Schrödinger, New York, USA), whose RMSD data are available in the Supporting Information (Figure S25).

## 3 Results

**FIGURE 2:**
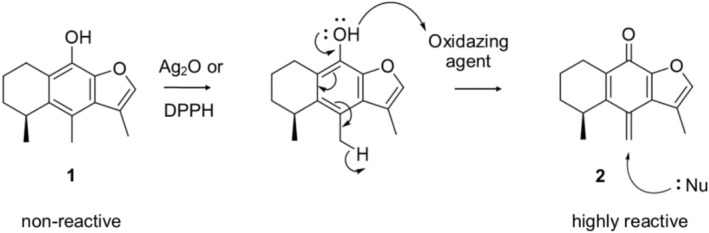
Oxidation of cacalol (**1**) to yield methylenecyclohexadienone (**2**) which increases its reactivity toward nucleophilic attacks at the exocyclic methylene group.

Oxidation of cacalol (**1**) using Ag_2_O in CH_2_Cl_2_ was an efficient and selective method to generate the methylenecyclohexadienone **2** in high yields (ca. 90%). Given that the oxidation reaction occurs in heterophase, its yield highly depends on the particle size of Ag_2_O, which is proportional to the contact surface between the Ag_2_O and cacalol (**1**). Parallelly, 2,2‐diphenyl‐1‐picrylhydrazyl (DPPH), an oxidizing, stable, and widely characterized free radical, was used to oxidize cacalol (**1**), thus emulating an exacerbated free radical environment as those present in cancer cells. Our results demonstrate that treatment of cacalol (**1**) with either Ag_2_O or DPPH generates **2**. Despite both oxidation methodologies used in this work affording **2** (Figure 2), full removal of unreacted DPPH from the reaction medium proved challenging to isolate **2** as a pure product. Therefore, the subsequent experiments were done using MTC (**2**) generated by the oxidation of cacalol (**1**) with Ag_2_O.

### 3.1 Competitive Covalent Reaction Between MTC (2) and Amino Acid Derivatives

Previous studies have demonstrated the high reactivity of methylene exocyclic moieties to generate covalent interactions with sidechain atoms of cysteine, lysine, and serine derivatives (Erbay et al. 2021; Pettinger et al. 2017; Wang et al. 2021). In this regard, and to demonstrate the reactivity of the methylene exocyclic group of **2**, this compound was treated with protected cysteine, lysine, and serine derivatives, which yielded compounds **4**, **5**, and **6** (Figure 1), respectively. In agreement with previous reports, these results show that compound **2** can form adducts with amino acids bearing heteroatoms in their side chains (e.g., cysteine, lysine, and serine), although with a very different reactivity. Thus, compound **2** exhibits a high capacity to react with the cysteine derivative (*N*‐Boc‐L‐Cys‐*O*Me) to generate **4** (yield 49% in 3 h), followed by the lysine derivative (*N*‐Boc‐L‐Lys‐*O*Me•HCl) to afford **5** (yield 9% in 24 h), and with more difficulty with the serine derivative (*N*‐Boc‐L‐Ser‐*O*Me) to give **6** (yield 5% in 150 h). Interestingly, compounds **4** and **6** are quite stable, in contrast to compound **5**, which resulted to be somewhat unstable, showing reversibility of the reaction during the purification process.

**FIGURE 3:**
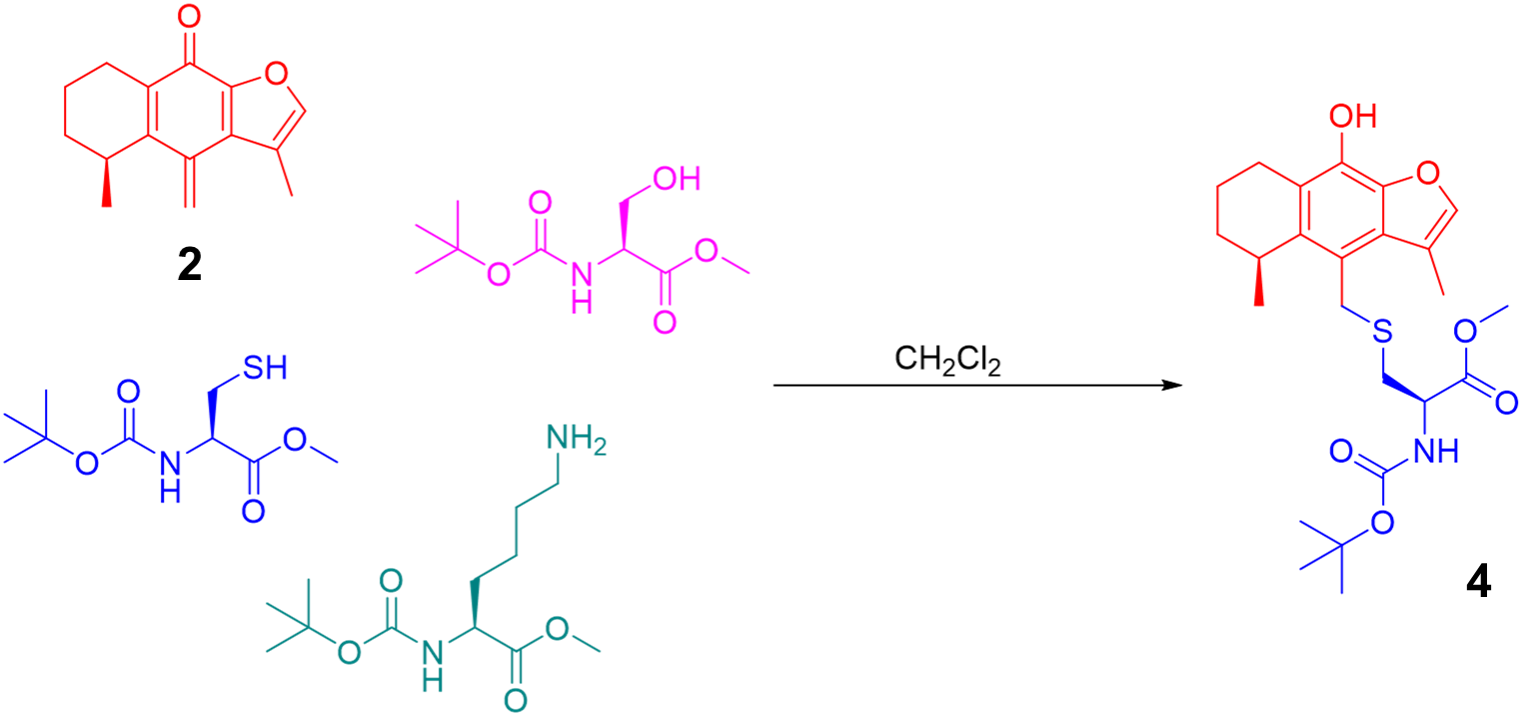
Reaction of **2** (red) with the three amino acid derivatives *N*‐Boc‐L‐Cys‐*O*Me (blue), *N*‐Boc‐L‐Lys‐*O*Me (green), and *N*‐Boc‐L‐Ser‐*O*Me (purple) all together in equimolar concentrations to yield **4** as the only product. This experiment demonstrates the high selectivity of the reaction between the sulfur atom of the cysteine derivative and the exocyclic methylene group of **2**.

Additionally, to verify the selectivity of the methylene exocyclic group of **2**, this compound was treated with the cysteine, lysine, and serine derivatives all together in equimolar concentrations with respect to **2** (Section 2.3), forming compound **4** as the only product. This experiment confirmed the high selectivity of **2** for generating the adduct with the cysteine derivative (*N*‐Boc‐L‐Cys‐*O*Me) as represented in Figure 3.

### 3.2 Tubulin Polymerization Assays

**FIGURE 4:**
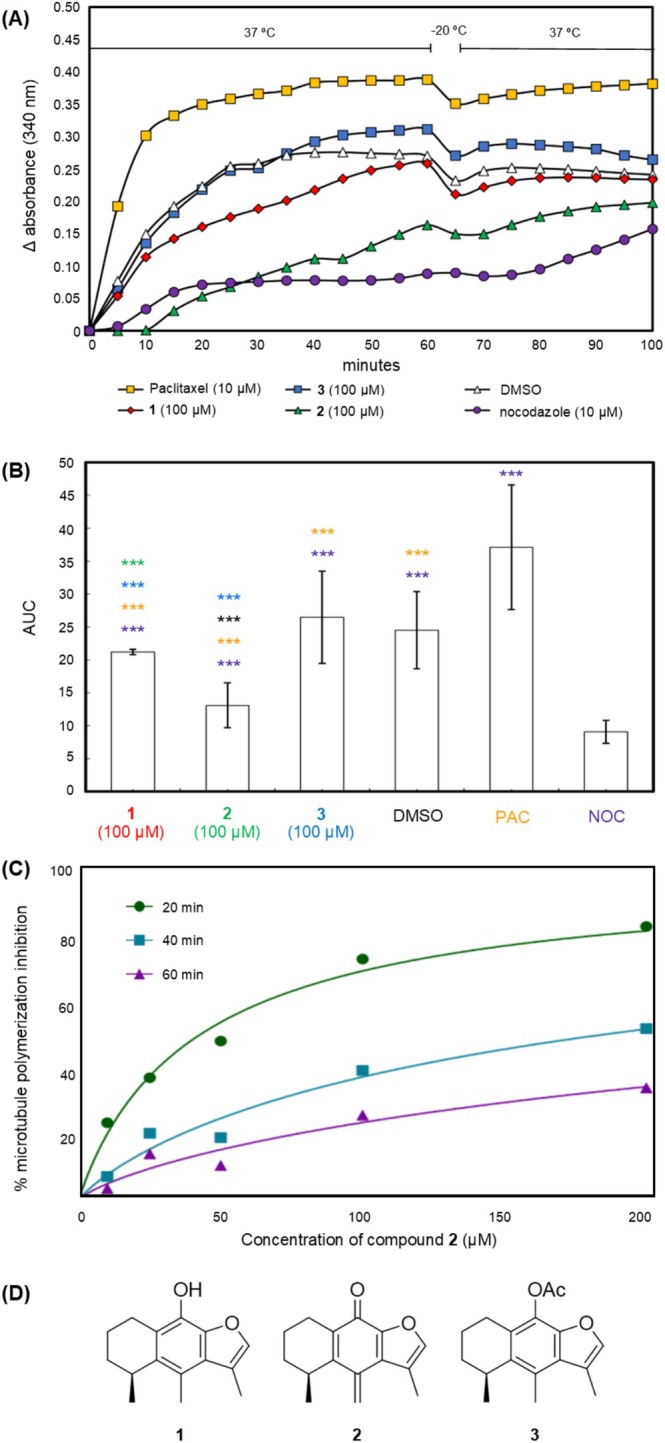
Tubulin polymerization activity of cacalol and derivatives. (A) Time‐dependent tubulin polymerization assay of cacalol (**1**), MTC (**2**), and cacalol acetate (**3**). (B) Comparison of the area under the tubulin polymerization curves of **1**–**3**, showing the *p* values < 0.001 (***). (C) Time‐dependent dose–response curves of tubulin polymerization assays of MTC (**2**). The data were determined at 20, 40, and 60 min, giving IC_50_ values of 43, 184, and 467 μM, respectively. (D) Chemical structures of evaluated compounds. All tests were carried out in triplicate.

Figure 4A,B, show the activity of **1**–**3** on tubulin polymerization assays (see Section 2.5) using a single dose (100 μM). The most active compound MTC (**2**) was studied using five doses during 60 min to evaluate the dose‐ and time‐dependent activity, which allowed us to quantify its inhibitory effect as IC_50_ values, resulting in 43 μM at 20 min, 184 μM at 40 min, and 467 μM at 60 min, using the software GraphPad Prism v.9.

### 3.3 Mass Spectrometry

**FIGURE 5:**
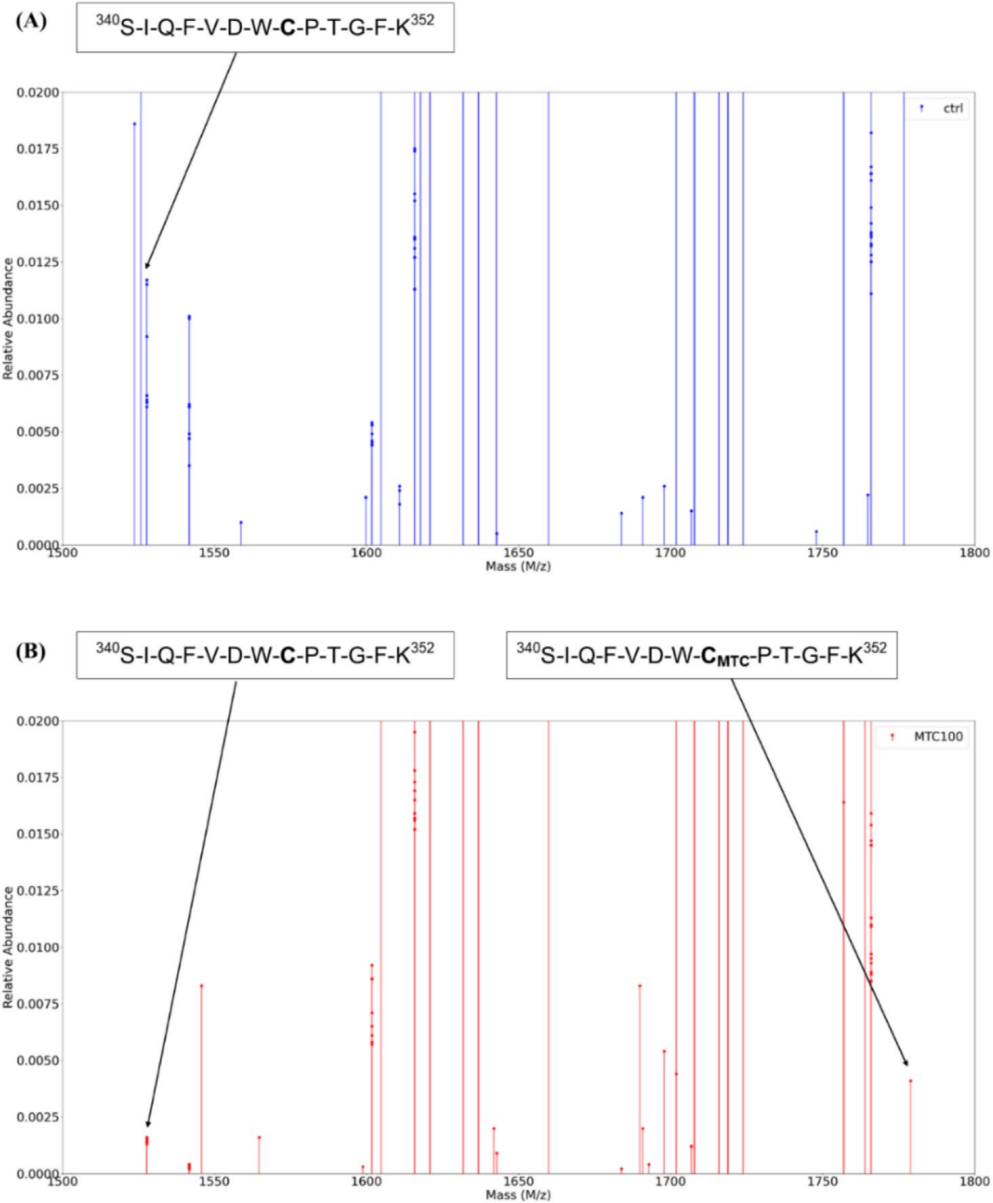
Mass spectrometry electrospray ionization data of (A) trypsinized tubulin and (B) trypsinized tubulin treated with 100 nM of MTC (**2**). Both panels are zoomed in the 1500–1800 *m*/*z* region. Panel A shows the peak at *m*/*z* 1527.73 corresponding to the peptide [^340^SIQFVDWCPTGFK^352^ + H]^+^ of α‐tubulin that contains Cys347. Panel B shows a peak at *m*/*z* 1778.86, which corresponds to the modified peptide with a covalently incorporated molecule of MTC (**2**) and a sodium atom ([M + MTC (**2**) + Na]^+^).

Mass spectrometry experiments were performed to determine the covalent binding site of MTC (**2**) on tubulins. Purified α‐β tubulin dimer and MTC (**2**) (1 and 100 μM) were incubated, followed by the removal of the quenched MTC (**2**) via SDS‐PAGE. The mass spectrometry results of the digested tubulins showed a covalent addition in the [^340^SIQFVDWCPTGFK^352^ + H]^+^ peptide (1527 Da) of α‐tubulin, which exhibited a molecular weight increase of 251 Da corresponding to MTC (**2**) + Na^+^ (228 + 23 Da); see in Figure 5. These results confirm the covalent binding interaction between α‐tubulin and MTC (**2**).

Interestingly, the identified peptide contains a cysteine residue (Cys347), an amino acid capable of forming adducts with the MTC (**2**) at a high selective rate (Figure 3). Namely, our results demonstrate that the MTC (**2**) interacts with the Cys347, a highly conserved amino acid at α‐tubulin isoforms (Hausrat et al. 2021).

### 3.4 Molecular Modeling

**FIGURE 6:**
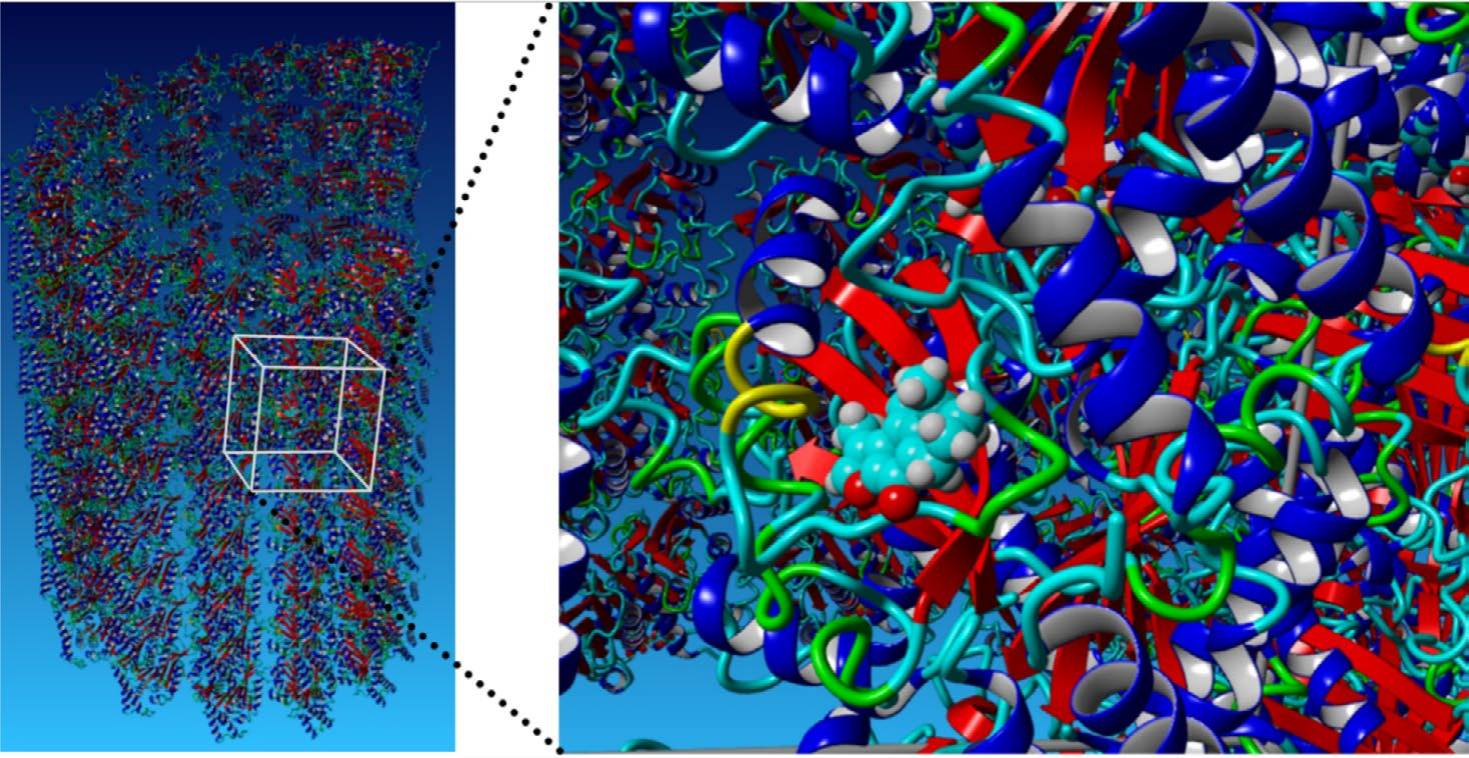
Molecular docking interactions between MTC (**2**) and the α‐tubulin in a microtubule fragment.

Blind molecular docking between MTC (**2**) and the PDB ID: 6O2S microtubule fragment (Figure 6) was performed using the Yasara software, which gave the binding scores of 25 poses within an energy range from 6.84 to 4.81 kcal/mol. In this software, more positive energies indicate stronger binding, and negative energies mean no binding. The output file with the binding scores and interactions is available in the Supporting Information (Appendix S1). The binding pose of MTC (**2**) at 5.5 kcal/mol displayed key interactions with Gln256, Thr257, Asn258, Leu259, Val260, Pro261, Tyr262, Met313, Ala314, Trp346, and Cys347 of α‐tubulin. This pose shows an internuclear distance of 3.9 Å between the C‐11 carbon atom of the methylene exocyclic group of MTC (**2**) and the sulfur atom of Cys347, which is very favorable for covalently connecting the two atoms. These findings are in full agreement with our experimental results.

**FIGURE 7:**
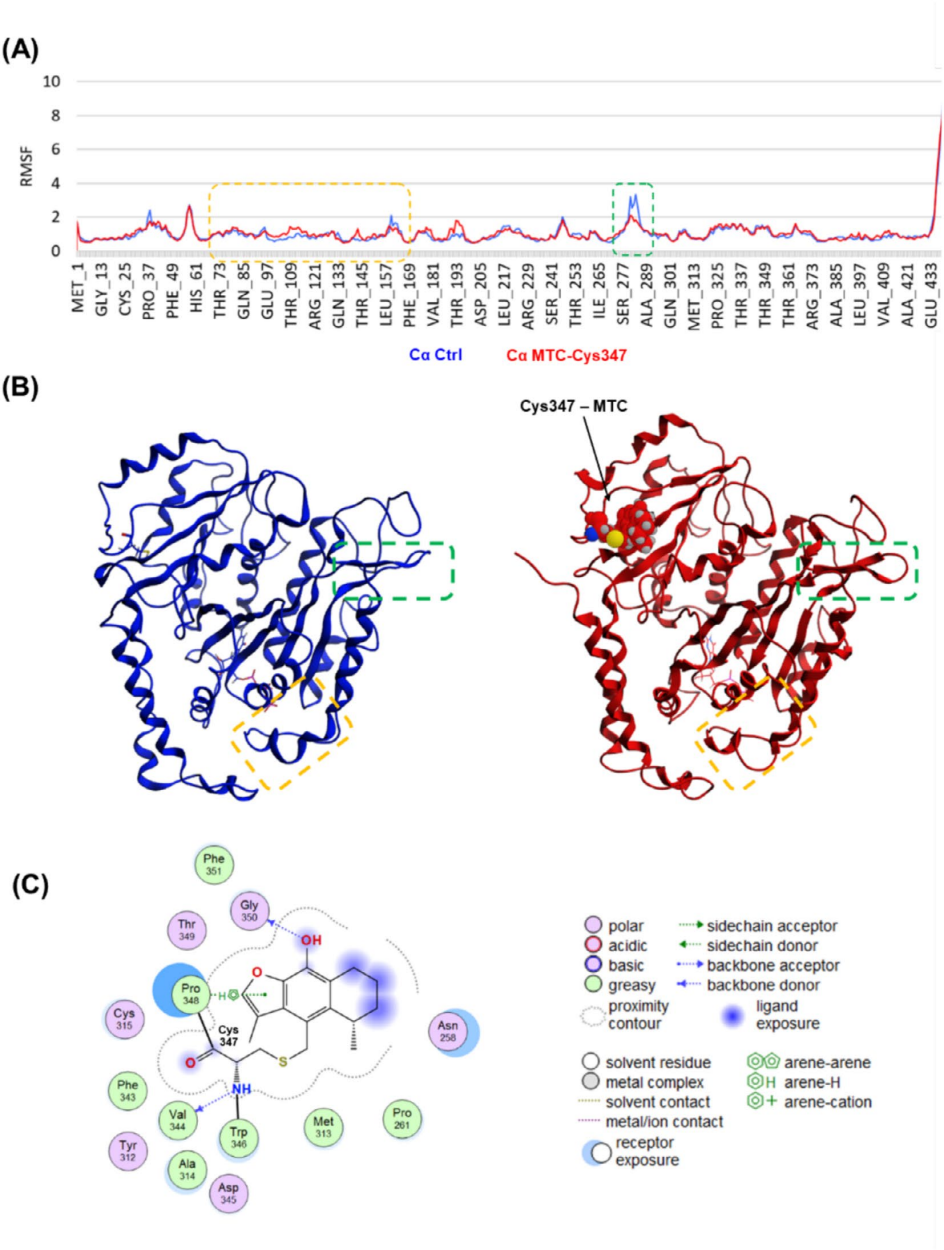
Molecular dynamics (MD) results of MTC (**2**) adduct with α‐tubulin. (A) RMSF values of the alpha carbon atoms for each amino acid with the MTC adduct (Cα MTC‐Cys347) during 150 ns of MD calculations in contrast to the native tubulin employed as the control (Cα Ctrl). Key regions with noticeable changes in RMSF values are marked with dotted green (M‐loop region from Pro274 to Gln285) and orange (GTP binding site) boxes. (B) MD final poses of α‐tubulin without and with the MTC adduct. (C) α‐tubulin‐MTC key interactions observed in the final pose of MD.

An in silico model of the adduct between α‐tubulin and the MTC (**2**) attached at Cys347 was constructed to explore the putative conformational changes observed in the protein when the covalent inhibitor is attached (Figure 7).

After MD calculations of the α‐tubulin‐MTC adduct at Cys347 (Figure 7), the complex undergoes conformational changes in a specific region of the GTP catalytic binding site (from Gly95 to Gly146; RMSF 0.14), which indicates a relevant movement in this region. Also, the M‐loop region shows noticeable conformational fluctuations (from Pro274 to Gln285; RMFS 0.39), which implies significant movements in this region. Additionally, Figure 7C shows the non‐covalent key interactions generated among MTC (**2**) and the neighboring amino acids (e.g., Pro348 and Gly350).

## 4 Discussion

Cacalol (**1**) is a natural product with selective inhibitory activity against cancer cell lines and tumors (Liu et al. 2010), which is associated with high ROS concentration, in contrast to normal cells that are linked with a balanced ROS concentration. However, the molecular mechanism through which cacalol generates these biological events has not been discussed previously. Additionally, cacalol has been associated with the selective modulation of the Akt‐SREBP‐FAS signaling pathway, which conducts apoptotic events by the activation of caspase‐3 (Liu et al. 2010). Interestingly, these same biological events were observed when tubulin polymerization inhibitors were tested. Namely, the relationships among the cacalol activity, the ROS concentration in a cellular context, and its molecular apoptotic mechanism, similarly to other tubulin polymerization inhibitors, have been perceived (Yan et al. 2021; Jackson and Singletary 2004).

As shown in Figure 2, cacalol (**1**) is a modulable compound that interacts with oxidant agents, generating a derivative with a reactive exocyclic methylenecyclohexadienone moiety (**2**) that could generate covalent adducts, for example, with reactive amino acids like cysteine (with the non‐shared electron pairs of the sulfur atom), lysine (with the non‐shared electron pair of the nitrogen atom), or serine (with the non‐shared electron pairs of the oxygen atom). These kinds of interactions have been documented in previous works focused on identifying and developing some covalent drugs (Lu et al. 2021; Fischer et al. 2020). The results presented in this work demonstrate that MTC (**2**) has a high affinity for the sulfur atom of cysteine derivatives. In the Tub‐Mts system, there are covalent inhibitors that have been associated with different binding sites, either in α or β tubulins (López‐López et al. 2021). A key example is pironetin, which interacts with α‐tubulin to inhibit the polymerization of microtubules (Yang et al. 2016; Prota et al. 2016). Pironetin generates a covalent bond by a Michael addition reaction between the *sp* ^2^ carbon atom at position 3 and the Cys316 of α‐tubulin, demonstrating its applicability for the development of tubulin covalent inhibitors.

**FIGURE 8:**
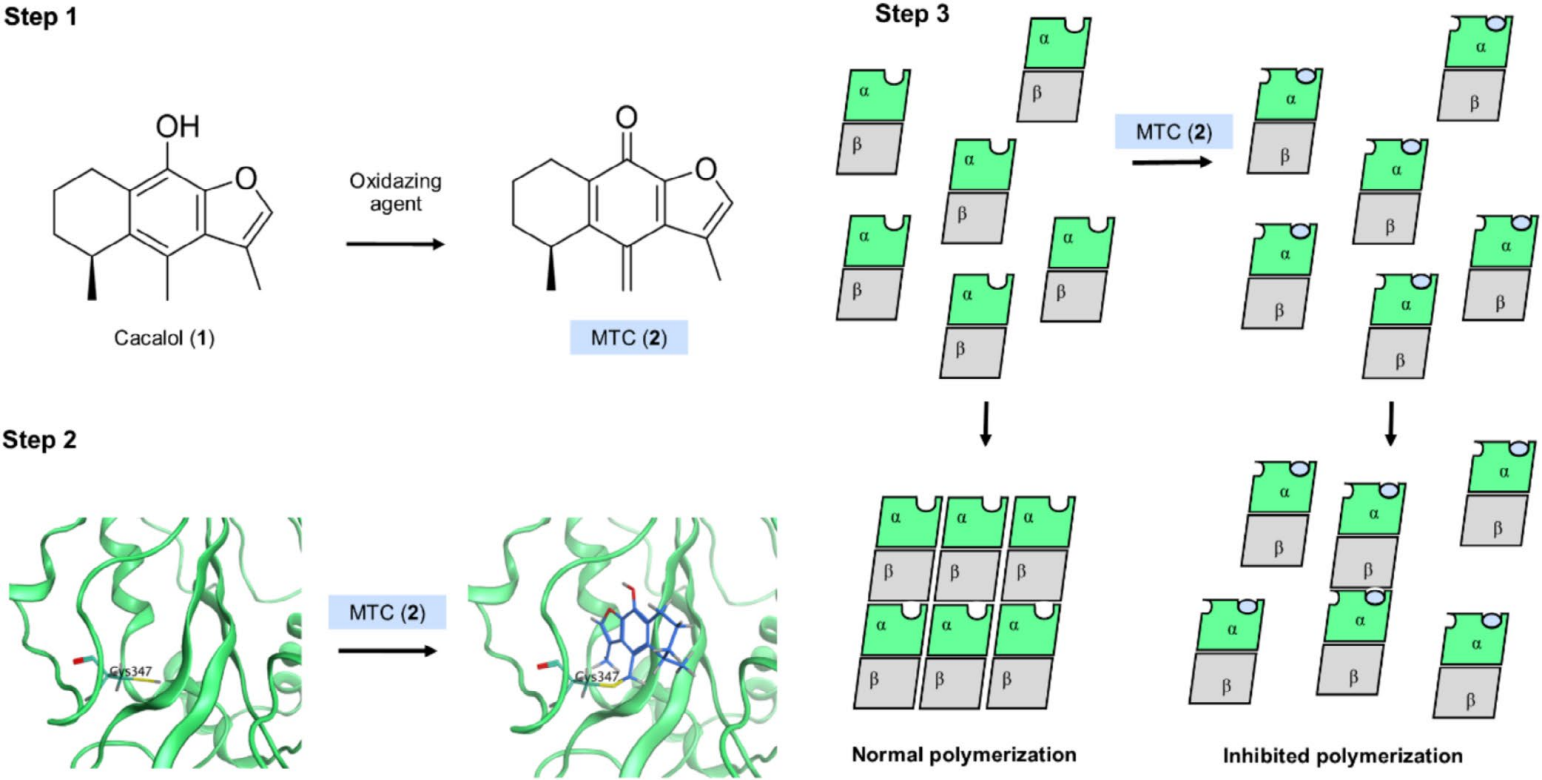
Molecular binding mechanism of cacalol (**1**)/MTC (**2**) related to their anti‐polymerization tubulin activity. Step 1: Cacalol (**1**) is transformed to MTC (**2**) in an oxidant medium; Step 2: MTC (**2**) binds covalently to Cys347 of α‐tubulin; Step 3: MTC‐α‐tubulin adduct reduces the polymerization rate of microtubules.

Figures 2 and 8 show that MTC (**2**) is an oxidized product of cacalol (**1**), which is distinguished by the presence of an exocyclic methylene group. MTC (**2**) is considered a highly reactive substance that may form a carbon–carbon bond by a Michael addition reaction with amino acids bearing heteroatoms in their side chains like cysteine. Furthermore, MTC (**2**) has the exceptional advantage that its exocyclic methylene group is formed when cacalol (**1**) is exposed to a highly oxidative environment such as that present in cancer cells, which are characterized by high levels of ROS. This phenomenon was mimicked by treatment of cacalol (**1**) with a stable free radical environment produced by a reaction medium containing DPPH (Section 2.4). Namely, the covalent mechanism of MTC (**2**) is ROS‐dependent, which could explain the selective activity of cacalol (**1**) against cancer cell lines and tumors, in contrast to healthy cell lines and normal biological environments (Liu et al. 2010).

It was found that the activity of MTC (**2**) is time‐dependent when the data set was separated according to the registration time (IC_50_ values of 43 μM at 20 min, 184 μM at 40 min, and 467 μM at 60 min). This phenomenon occurs when reactive compounds also interact with other molecules, particularly with the solvent, reducing their initial concentration through time. This has been studied for other covalent bioactive compounds that are sensitive to the media. An illustrative example of these kinds of compounds is the NF‐κB inhibitor parthenolide (Hotta et al. 2021), whose methylene exocyclic group reacts with water molecules (Prieto 2022), reducing the in vitro activity through time. Covalent inhibitors with moderate in vitro IC_50_ values can still be pharmacologically valuable (Schaefer and Cheng 2023). Their irreversible binding allows transient exposure to yield long‐lasting effects, offering the potential for intermittent dosing regimens that limit systemic toxicity.

The MD results presented herein show that the presence of the Cys347‐MTC adduct increases the mobility in the M‐loop region and reduces the flexibility in the GTP binding site region in α‐tubulin. Interestingly, existing biophysics reports demonstrate that changes in the movement of these regions generate microtubule destabilizations, promoting inhibition of the tubulin polymerization (Mitra and Sept 2008) in agreement with our experimental results (Figure 4). Figure 8 shows an overview of the cacalol (**1**)/MTC (**2**) inhibitory binding mechanism against tubulin based on the result of this work.

Previous reports have studied the reactivity of Cys347 in α‐tubulin, demonstrating that this amino acid is related to post‐translational modifications involved in the stability of the Tub–Mts system. Also, Cys347 was identified as the most reactive cysteine in tubulin (Britto et al. 2002), which fully agrees with our results. Some reports suggest that the covalent bonding of small molecules at Cys347 generates a significant reduction in microtubule stability and polymerization rate (Piroli et al. 2014; Stewart et al. 2007). The preservation of tubulin integrity is crucial for maintaining its functions, while mutations in many regions may cause distinct defects in microtubule functions (Aiken et al. 2019). Additionally, in vitro models confirm that the covalent modification of Cys347 is associated with increased apoptotic events (Mi and Chung 2008; Han et al. 2010). Interestingly, these observations agree with the results of evaluations of cacalol (**1**) on its pro‐apoptotic and anti‐cancer activities (Liu et al. 2010). It is recognized that several covalent inhibitors may exhibit target promiscuity by forming irreversible bonds with cysteine residues on proteins beyond tubulin. For example, the natural product parthenolide was initially discovered as a tyrosinase inhibitor, and subsequent studies confirmed its complementary and covalent activity in tubulin (Hotta et al. 2021). We underscore that covalent tubulin modifiers, while effective in impairing microtubule dynamics, frequently lack perfect specificity and may engage unintended biological targets. In this regard, this work presents the first rational mechanism of action at the molecular level that explains the reported activity of cacalol derivatives. However, alternative targets must be studied for a complete understanding of their possible promiscuity.

Although the methylene group is commonly used in the design of covalent inhibitors (Erbay et al. 2021; Tuley and Fast 2018), this is the first report of a chemical structure, namely cacalol (**1**), that is capable of being converted into a highly reactive compound bearing an exocyclic methylene group (**2**), promoted by high ROS levels. This opens new perspectives on the design of covalent inhibitors against different kinds of diseases characterized by high ROS levels, like cancer, as well as immunologic and metabolic diseases (Ranneh et al. 2017).

## 5 Conclusions

The mechanism described in this work is promoted by an oxidated form of cacalol (**1**), the MTC (**2**), which reacts at the exocyclic methylene group with the sulfur atom of Cys347 in α‐tubulin. The covalent binding mechanism was demonstrated using chemical reactivity, mass spectrometry, in vitro tubulin polymerization, and in silico modeling approaches. This is the first report of a modulable, ROS‐dependent bioactive molecule that could open new perspectives on the development of covalent inhibitors. Finally, these results promote the use of the cacalol scaffold to generate novel, selective, and modulable anti‐cancer agents. Some perspectives of this work are to develop novel cacalol (**1**) derivatives, study their potency and safety using additional in vitro or in vivo studies, and use crystallographic methods to explore the conformational changes of α‐tubulin in the presence of MTC (**2**).

## Conflicts of Interest

The authors declare no conflicts of interest.

## Supporting information


**Data S1:** cbdd70165‐sup‐0001‐Supinfo.docx.

## Data Availability

The data that supports the findings of this study are available in the Supporting Information of this article.

## References

[cbdd70165-bib-0001] Aiken, J. , J. K. Moore , and E. A. Bates . 2019. “TUBA1A Mutations Identified in Lissencephaly Patients Dominantly Disrupt Neuronal Migration and Impair Dynein Activity.” Human Molecular Genetics 28, no. 8: 1227–1243. 10.1093/hmg/ddy416. 30517687 PMC6452179

[cbdd70165-bib-0002] Alarcon‐Aguilar, F. J. , A. Fortis‐Barrera , S. Angeles‐Mejia , et al. 2010. “Anti‐Inflammatory and Antioxidant Effects of a Hypoglycemic Fructan Fraction From *Psacalium peltatum* (HBK) Cass. In Streptozotocin‐Induced Diabetes Mice.” Journal of Ethnopharmacology 132, no. 2: 400–407. 10.1016/j.jep.2010.08.003. 20713141

[cbdd70165-bib-0003] Anaya, A. L. , B. E. Hernández‐Bautista , A. Torres‐Barragán , J. León‐Cantero , and M. Jiménez‐Estrada . 1996. “Phytotoxicity of Cacalol and Some Derivatives Obtained From the Roots of *Psacalium decompositum* (A. Gray) H. Rob. & Brettell (Asteraceae), Matarique or Maturin.” Journal of Chemical Ecology 22: 393–403. 10.1007/BF02033643. 24227480

[cbdd70165-bib-0004] Britto, P. J. , L. Knipling , and J. Wolff . 2002. “The Local Electrostatic Environment Determines Cysteine Reactivity of Tubulin.” Journal of Biological Chemistry 277, no. 32: 29018–29027. 10.1074/jbc.M204263200. 12023292

[cbdd70165-bib-0005] Castillo‐Arellano, J. I. , J. C. Gómez‐Verjan , N. A. Rojano‐Vilchis , et al. 2018. “Chemoinformatic Analysis of Selected Cacalolides From *Psacalium decompositum* (A. Gray) H. Rob. & Brettell and *Psacalium peltatum* (Kunth) Cass. And Their Effects on FcεRI‐Dependent Degranulation in Mast Cells.” Molecules 23, no. 12: 3367. 10.3390/molecules23123367. 30572603 PMC6321304

[cbdd70165-bib-0006] Chemical Computing Group Inc . 2023. Molecular Operating Environment (MOE). Chemical Computing Group Inc.

[cbdd70165-bib-0007] del Río, R. E. , J. C. Pardo‐Novoa , C. M. Cerda‐García‐Rojas , and P. Joseph‐Nathan . 2021. “Vibrational Circular Dichroism Behavior of Quinol Cacalolides From *Psacalium aff. sinuatum* .” Journal of Molecular Structure 1224: 128987. 10.1016/j.molstruc.2020.128987.

[cbdd70165-bib-0008] Erbay, T. G. , D. P. Dempe , B. Godugu , P. Liu , and K. M. Brummond . 2021. “Thiol Reactivity of N‐Aryl α‐Methylene‐γ‐Lactams: A Reactive Group for Targeted Covalent Inhibitor Design.” Journal of Organic Chemistry 86, no. 17: 11926–11936. 10.1021/acs.joc.1c01335. 34379423

[cbdd70165-bib-0009] Eshun‐Wilson, L. , R. Zhang , D. Portran , et al. 2019. “Effects of α‐Tubulin Acetylation on Microtubule Structure and Stability.” Proceedings of the National Academy of Sciences of the United States of America 116, no. 21: 10366–10371. 10.1073/pnas.1900441116. 31072936 PMC6535015

[cbdd70165-bib-0010] Fischer, L. , R. C. Steffens , T. J. Paul , and L. Hartmann . 2020. “Catechol‐Functionalized Sequence‐Defined Glycomacromolecules as Covalent Inhibitors of Bacterial Adhesion.” Polymer Chemistry 11, no. 37: 6091–6096. 10.1039/D0PY00975J.

[cbdd70165-bib-0011] Gómez‐Vidales, V. , G. Granados‐Oliveros , A. Nieto‐Camacho , M. Reyes‐Solís , and M. Jiménez‐Estrada . 2014. “Cacalol and Cacalol Acetate as Photoproducers of Singlet Oxygen and as Free Radical Scavengers, Evaluated by EPR Spectroscopy and TBARS.” RSC Advances 4, no. 3: 1371–1377. 10.1039/C3RA42848F.

[cbdd70165-bib-0012] Han, H. , Y. Zhao , T. Cuthbertson , R. F. Hartman , and S. D. Rose . 2010. “Cell Cycle Arrest and Apoptosis Induction by an Anticancer Chalcone Epoxide.” Archiv der Pharmazie 343, no. 8: 429–439. 10.1002/ardp.200900261. 20726006

[cbdd70165-bib-0013] Hausrat, T. J. , J. Radwitz , F. L. Lombino , P. Breiden , and M. Kneussel . 2021. “Alpha‐ and Beta‐Tubulin Isotypes Are Differentially Expressed During Brain Development.” Developmental Neurobiology 81, no. 3: 333–350. 10.1002/dneu.22745. 32293117

[cbdd70165-bib-0014] Hee‐Jung, K. , H. Sura , Y. L. Hee , and L. Kong‐Joo . 2015. “ROSics: Chemistry and Proteomics of Cysteine Modifications in Redox Biology.” Mass Spectrometry Reviews 34, no. 2: 184–208. 10.1002/mas.21430. 24916017 PMC4340047

[cbdd70165-bib-0015] Hotta, T. , S. E. Haynes , T. L. Blasius , et al. 2021. “Parthenolide Destabilizes Microtubules by Covalently Modifying Tubulin.” Current Biology 31, no. 4: 900–907. 10.1016/j.cub.2020.11.055. 33482110 PMC7931505

[cbdd70165-bib-0016] Inman, W. D. , J. Luo , S. D. Jolad , S. R. King , and R. Cooper . 1999. “Antihyperglycemic Sesquiterpenes From *Psacalium decompositum* .” Journal of Natural Products 62, no. 8: 1088–1092. 10.1021/np990023v. 10479309

[cbdd70165-bib-0017] Jackson, S. J. , and K. W. Singletary . 2004. “Sulforaphane: A Naturally Occurring Mammary Carcinoma Mitotic Inhibitor, Which Disrupts Tubulin Polymerization.” Carcinogenesis 25, no. 2: 219–227. 10.1093/carcin/bgg192. 14578157

[cbdd70165-bib-0018] Janke, C. , and M. M. Magiera . 2020. “The Tubulin Code and Its Role in Controlling Microtubule Properties and Functions.” Nature Reviews Molecular Cell Biology 21, no. 6: 307–326. 10.1038/s41580-020-0214-3. 32107477

[cbdd70165-bib-0019] Jimenez‐Estrada, M. , R. R. Chilpa , T. R. Apan , et al. 2006. “Anti‐Inflammatory Activity of Cacalol and Cacalone Sesquiterpenes Isolated From *Psacalium decompositum* .” Journal of Ethnopharmacology 105, no. 1–2: 34–38. 10.1016/j.jep.2005.09.039. 16307855

[cbdd70165-bib-0020] Krieger, E. , and G. Vriend . 2014. “YASARA View—Molecular Graphics for All Devices—From Smartphones to Workstations.” Bioinformatics 30, no. 20: 2981–2982. 10.1093/bioinformatics/btu426. 24996895 PMC4184264

[cbdd70165-bib-0022] Liu, W. , E. Furuta , K. Shindo , et al. 2010. “Cacalol, a Natural Sesquiterpene, Induces Apoptosis in Breast Cancer Cells by Modulating Akt‐SREBP‐FAS Signaling Pathway.” Breast Cancer Research and Treatment 128: 57–68. 10.1007/s10549-010-1076-8. 20665104

[cbdd70165-bib-0023] López‐López, E. , C. M. Cerda‐García‐Rojas , and J. L. Medina‐Franco . 2021. “Tubulin Inhibitors: A Chemoinformatic Analysis Using Cell‐Based Data.” Molecules 26, no. 9: 2483. 10.3390/molecules26092483. 33923169 PMC8123128

[cbdd70165-bib-0024] López‐López, E. , C. M. Cerda‐García‐Rojas , and J. L. Medina‐Franco . 2023. “Consensus Virtual Screening Protocol Towards the Identification of Small Molecules Interacting With the Colchicine Binding Site of the Tubulin‐Microtubule System.” Molecular Informatics 42, no. 1: 2200166. 10.1002/minf.202200166. 36175374 PMC10078098

[cbdd70165-bib-0025] Lu, X. , J. B. Smaill , A. V. Patterson , and K. Ding . 2021. “Discovery of Cysteine‐Targeting Covalent Protein Kinase Inhibitors.” Journal of Medicinal Chemistry 65, no. 1: 58–83. 10.1021/acs.jmedchem.1c01719. 34962782

[cbdd70165-bib-0026] Mi, L. , and F. L. Chung . 2008. “Binding to Protein by Isothiocyanates: A Potential Mechanism for Apoptosis Induction in Human Nonsmall Lung Cancer Cells.” Nutrition and Cancer 60, no. S1: 12–20. 10.1080/01635580802381287. 19003576

[cbdd70165-bib-0027] Mitra, A. , and D. Sept . 2008. “Taxol Allosterically Alters the Dynamics of the Tubulin Dimer and Increases the Flexibility of Microtubules.” Biophysical Journal 95, no. 7: 3252–3258. 10.1529/biophysj.108.133884. 18621813 PMC2547448

[cbdd70165-bib-0028] Mora‐Ramiro, B. , M. Jiménez‐Estrada , A. Zentella‐Dehesa , et al. 2020. “Cacalol Acetate, a Sesquiterpene From *Psacalium decompositum*, Exerts an Anti‐Inflammatory Effect Through LPS/NF‐KB Signaling in Raw 264.7 Macrophages.” Journal of Natural Products 83, no. 8: 2447–2455. 10.1021/acs.jnatprod.0c00300. 32672964

[cbdd70165-bib-0029] O'Boyle, N. M. , M. Banck , C. A. James , C. Morley , T. Vandermeersch , and G. R. Hutchison . 2011. “Open Babel: An Open Chemical Toolbox.” Journal of Cheminformatics 3: 1–14. 10.1186/1758-2946-3-33. 21982300 PMC3198950

[cbdd70165-bib-0030] Omura, K. , M. Nakanishi , K. Takai , and K. Naya . 1978. “The Sesquiterpenes of Cacalia Species: 8‐Oxocacalol and the Stereochemistry of Cacalone Epimers.” Chemistry Letters 7, no. 11: 1257–1260. 10.1246/cl.1978.1257.

[cbdd70165-bib-0031] Pettinger, J. , K. Jones , and M. D. Cheeseman . 2017. “Lysine‐Targeting Covalent Inhibitors.” Angewandte Chemie International Edition 56, no. 48: 15200–15209. 10.1002/anie.201707630. 28853194

[cbdd70165-bib-0032] Piroli, G. G. , A. M. Manuel , M. D. Walla , et al. 2014. “Identification of Protein Succination as a Novel Modification of Tubulin.” Biochemical Journal 462, no. 2: 231–245. 10.1042/BJ20131581. 24909641 PMC4324573

[cbdd70165-bib-0033] Prieto, J. M. 2022. “Stability of Feverfew and Its Active Principle Parthenolide: An Elusive Antimigraine Herbal Medicine.” Journal of Natural Products Discovery 1, no. 1: 659. 10.24377/jnpd.article659.

[cbdd70165-bib-0034] Prota, A. E. , J. Setter , A. B. Waight , et al. 2016. “Pironetin Binds Covalently to αCys316 and Perturbs a Major Loop and Helix of α‐Tubulin to Inhibit Microtubule Formation.” Journal of Molecular Biology 428, no. 15: 2981–2988. 10.1016/j.jmb.2016.06.023. 27395016

[cbdd70165-bib-0035] Ranneh, Y. , F. Ali , A. M. Akim , H. A. Hamid , H. Khazaai , and A. Fadel . 2017. “Crosstalk Between Reactive Oxygen Species and Pro‐Inflammatory Markers in Developing Various Chronic Diseases: A Review.” Applied Biological Chemistry 60: 327–338. 10.1007/s13765-017-0285-9.

[cbdd70165-bib-0036] Rostro‐Alonso, G. O. , A. I. Castillo‐Montoya , J. C. García‐Acosta , et al. 2024. “Cacalol Acetate as Anticancer Agent: Antiproliferative, Pro‐Apoptotic, Cytostatic, and Anti‐Migratory Effects.” Current Issues in Molecular Biology 46: 9298–9311. 10.3390/cimb46090550. 39329902 PMC11430360

[cbdd70165-bib-0037] Schaefer, D. , and X. Cheng . 2023. “Recent Advances in Covalent Drug Discovery.” Pharmaceuticals 16, no. 5: 663. 10.3390/ph16050663. 37242447 PMC10220821

[cbdd70165-bib-0038] Shindo, K. , M. Kimura , and M. Iga . 2004. “Potent Antioxidative Activity of Cacalol, a Sesquiterpene Contained in *Cacalia delphiniifolia* Sleb et Zucc.” Bioscience, Biotechnology, and Biochemistry 68, no. 6: 1393–1394. 10.1271/bbb.68.1393. 15215613

[cbdd70165-bib-0039] Silva‐García, E. M. , C. M. Cerda‐García‐Rojas , R. E. Del Río , and P. Joseph‐Nathan . 2019. “Parvifoline Derivatives as Tubulin Polymerization Inhibitors.” Journal of Natural Products 82, no. 4: 840–849. 10.1021/acs.jnatprod.8b00860. 30883116

[cbdd70165-bib-0040] Stewart, B. J. , J. A. Doorn , and D. R. Petersen . 2007. “Residue‐Specific Adduction of Tubulin by 4‐Hydroxynonenal and 4‐Oxononenal Causes Cross‐Linking and Inhibits Polymerization.” Chemical Research in Toxicology 20, no. 8: 1111–1119. 10.1021/tx700106v. 17630713

[cbdd70165-bib-0041] Tuley, A. , and W. Fast . 2018. “The Taxonomy of Covalent Inhibitors.” Biochemistry 57, no. 24: 3326–3337. 10.1021/acs.biochem.8b00315. 29689165 PMC6016374

[cbdd70165-bib-0042] Wang, L. , L. P. Riel , B. Bajrami , B. Deng , A. R. Howell , and X. Yao . 2021. “α‐Methylene‐β‐Lactone Scaffold for Developing Chemical Probes at the Two Ends of the Selectivity Spectrum.” Chembiochem 22, no. 3: 505–515. 10.1002/cbic.202000605. 32964640 PMC8114233

[cbdd70165-bib-0043] Winkler, C. , K. Denker , S. Wortelkamp , and A. Sickmann . 2007. “Silver‐ and Coomassie‐Staining Protocols: Detection Limits and Compatibility With ESI MS.” Electrophoresis 28, no. 12: 2095–2099. 10.1002/elps.200600670. 17516579

[cbdd70165-bib-0044] Yan, Y. , Y. Zhou , J. Li , et al. 2021. “Sulforaphane Downregulated Fatty Acid Synthase and Inhibited Microtubule‐Mediated Mitophagy Leading to Apoptosis.” Cell Death & Disease 12, no. 10: 917. 10.1038/s41419-021-04198-2. 34620841 PMC8497537

[cbdd70165-bib-0045] Yang, J. , Y. Wang , T. Wang , et al. 2016. “Pironetin Reacts Covalently With Cysteine‐316 of α‐Tubulin to Destabilize Microtubule.” Nature Communications 7, no. 1: 12103. 10.1038/ncomms12103. PMC4931326 27357539

[cbdd70165-bib-0046] Yuste, F. , E. Diaz , F. Walls , and K. Jankowski . 1976. “The Structure of Cacalone.” Journal of Organic Chemistry 41, no. 26: 4103–4106. 10.1021/jo00888a013.

