# Supplementary material for: A Covalent and Modulable Inhibitor of the Tubulin‐Microtubule System: Insights Into the Mechanism of Cacalol
Source: Chem Biol Drug Des. 2025 Sep 11;106(3):e70165. doi: 10.1111/cbdd.70165 (PMC12426469; doi:10.1111/cbdd.70165)
Supplement: Supplementary file 1 — Data S1: cbdd70165‐sup‐0001‐Supinfo.docx. [file CBDD-106-e70165-s001.docx]

Supporting Information

A covalent and modulable inhibitor of the tubulin-microtubule system: Insights into the mechanism of cacalol

Edgar López-López^1,2^, José L. Medina-Franco^2^, Eric Salinas-Arellano^1,3^, Karen J. Ardila-Fierro^1,4^, Julio C. Pardo-Novoa^1^, Rosa E. del Río^5^, and Carlos M. Cerda-García-Rojas^1,^*

^1^Departamento de Química y Programa de Posgrado en Farmacología, Centro de Investigación y de Estudios Avanzados del Instituto Politécnico Nacional, Av. Instituto Politécnico Nacional 2508, 07360 Mexico City, Mexico.

^2^DIFACQUIM Research Group, Department of Pharmacy, School of Chemistry, Universidad Nacional Autónoma de México, 04510 Mexico City, Mexico.

^3^Departamento de Ciencias de la Tierra y de la Vida, Centro Universitario de los Lagos, Universidad de Guadalajara, Enrique Díaz de León 1144, Paseos de la Montaña, Lagos de Moreno, 47463 Jalisco, Mexico.

^4^Grupo Ciencia de los Materiales, Instituto de Química, Facultad de Ciencias Exactas y Naturales, Universidad de Antioquia, Calle 70 No 52–21, Medellín, Colombia.

^5^Instituto de Investigaciones Químico-Biológicas, Universidad Michoacana de San Nicolás de Hidalgo, Ciudad Universitaria, Morelia, Michoacán 58040, Mexico.

INDEX

| **Material** | **Name** | **Page** |
| --- | --- | --- |
| Figure S1. | ^1^H NMR spectrum of cacalol (**1**). | 3 |
| Figure S1a. | Region of the ^1^H NMR spectrum (δ 3.20–1.10 ppm) of cacalol (**1**). | 4 |
| Figure S2. | ^13^C NMR spectrum of cacalol (**1**). | 5 |
| Figure S3. | UHPLC-DAD of cacalol (**1**). | 6 |
| Figure S4. | ^1^H NMR spectrum of methylenecyclohexadienone derivative (MTC) (**2**). | 7 |
| Figure S4a | Region of the ^1^H NMR spectrum (δ 3.20–1.20 ppm) of methylenecyclohexadienone derivative (MTC) (**2**). | 8 |
| Figure S5. | ^13^C NMR spectrum of methylenecyclohexadienone derivative (MTC) (**2**). | 9 |
| Figure S6. | HRESIMS of methylenecyclohexadienone derivative (MTC) (**2**). | 10 |
| Figure S7. | IR spectrum of methylenecyclohexadienone derivative (MTC) (**2**). | 11 |
| Figure S8. | HPLC-MS of methylenecyclohexadienone derivative (MTC) (**2**). | 12 |
| Figure S9. | ^1^H NMR spectrum of cacalol acetate (**3**). | 13 |
| Figure S9a. | Region of the ^1^H NMR spectrum (δ 3.30–1.15 ppm) of cacalol acetate (**3**). | 14 |
| Figure S10. | ^13^C NMR spectrum of cacalol acetate (**3**). | 15 |
| Figure S11. | HPLC-MS of cacalol acetate (**3**). | 16 |
| Figure S12. | ^1^H NMR spectrum of compound of **4**. | 17 |
| Figure S12a. | Region of the ^1^H NMR spectrum (δ 5.50–3.97 ppm) of **4**. | 18 |
| Figure S12b. | Region of the ^1^H NMR spectrum (δ 3.50–2.56 ppm) of **4**. | 19 |
| Figure S13. | ^13^C NMR spectrum of compound of **4**. | 20 |
| Figure S14. | HRESIMS of **4**. | 21 |
| Figure S15. | IR spectrum of **4**. | 22 |
| Figure S16. | ^1^H NMR spectrum of compound **5**. | 23 |
| Figure S16a | Region of the ^1^H NMR spectrum (δ 5.14–3.56 ppm) of **5**. | 24 |
| Figure S16b | Region of the ^1^H NMR spectrum (δ 3.40–2.20 ppm) of **5**. | 25 |
| Figure S17. | ^13^C NMR spectrum of compound **5**. | 26 |
| Figure S18. | HRESIMS of **5**. | 27 |
| Figure S19. | IR spectrum of **5**. | 28 |
| Figure S20. | ^1^H NMR spectrum of compound **6**. | 29 |
| Figure S20a | Region of the ^1^H NMR spectrum (*δ* 4.50–2.52 ppm) of **6**. | 30 |
| Figure S21. | ^13^C NMR spectrum of compound **6**. | 31 |
| Figure S22. | HRESIMS of **6**. | 32 |
| Figure S23. | IR spectrum of **6**. | 33 |
| Figure S24. | Tubulin polymerization curves of MTC (**2**). | 34 |
| Figure S25. | Molecular dynamics results of the complex α-tubulin-MTC. | 35 |
| Appendix S1. | Yasara output for global docking result analysis | 36 |


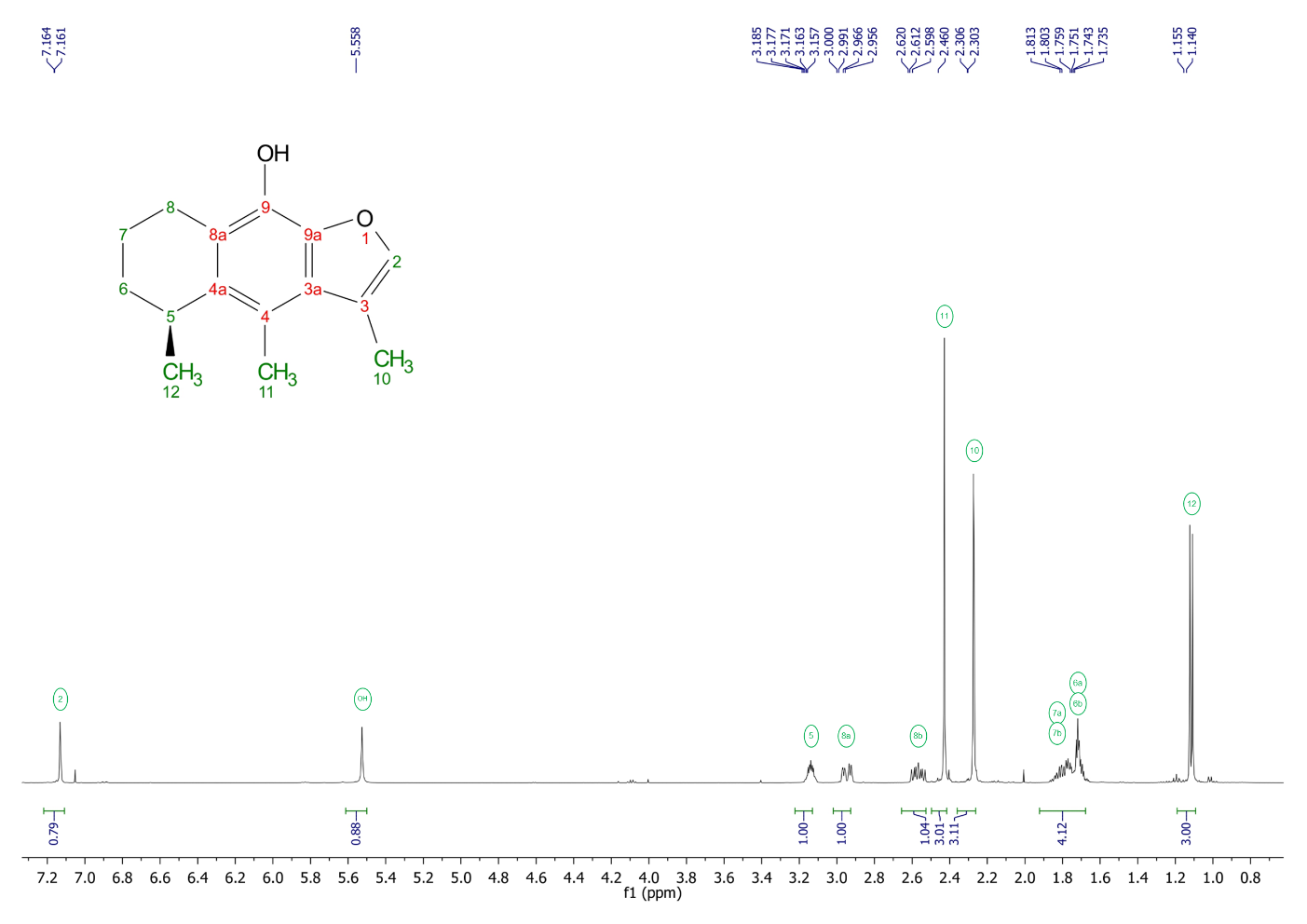
**Figure S1.** ^1^H NMR spectrum of cacalol (**1**) (500 MHz, CDCl_3_).


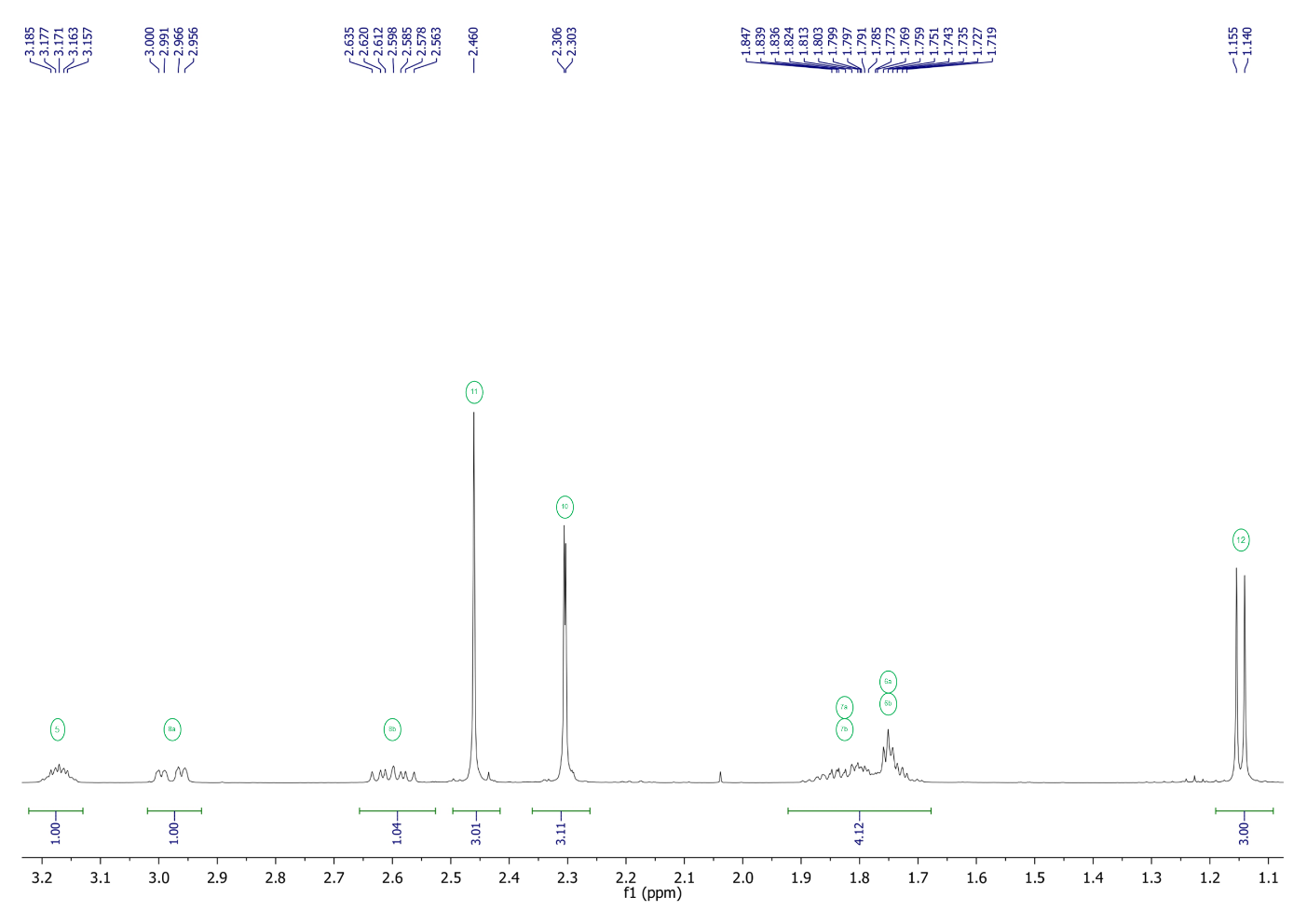
**Figure S1a.** Region of the ^1^H NMR spectrum (*δ* 3.20–1.10 ppm) of cacalol (**1**) (500 MHz, CDCl_3_).


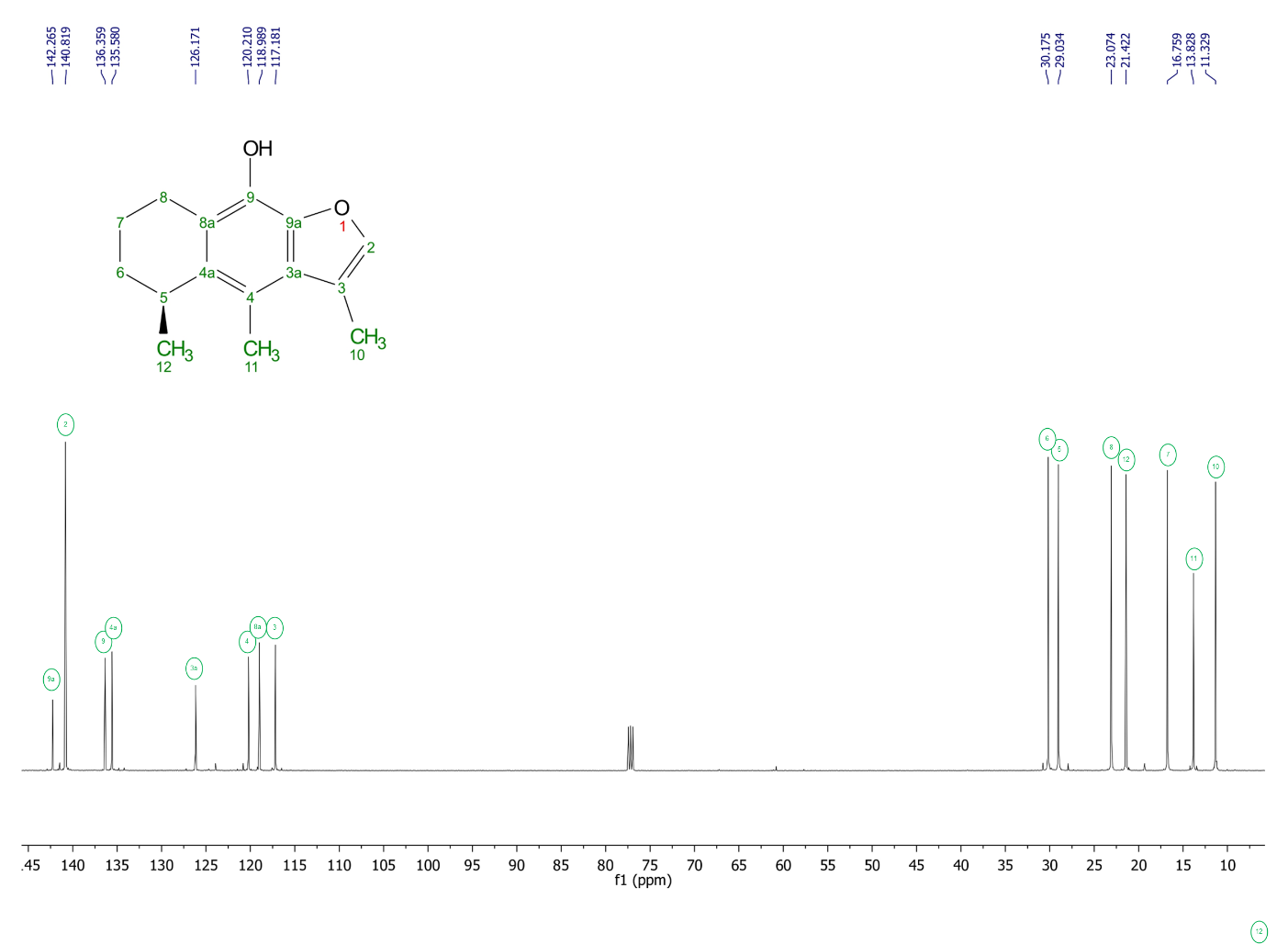


**Figure S2.** ^13^C NMR spectrum of cacalol (**1**) (125.8 MHz, CDCl_3_).

Peak results

| **#** | **Time (min)** | **Area** | **Height** | **Area (%)** | **Resolution** |
| --- | --- | --- | --- | --- | --- |
| 1 | 1.950 | 1.4958 | 11.552 | 0.71 | 5.52 |
| 2 | 3.270 | 205.5800 | 1221.838 | 97.82 | 12.90 |
| 3 | 6.283 | 1.5228 | 12.040 | 0.72 | 27.98 |
| 4 | 11.757 | 1.5621 | 12.352 | 0.74 | n.a |

**Figure S3.** UHPLC-DAD of cacalol (**1**).


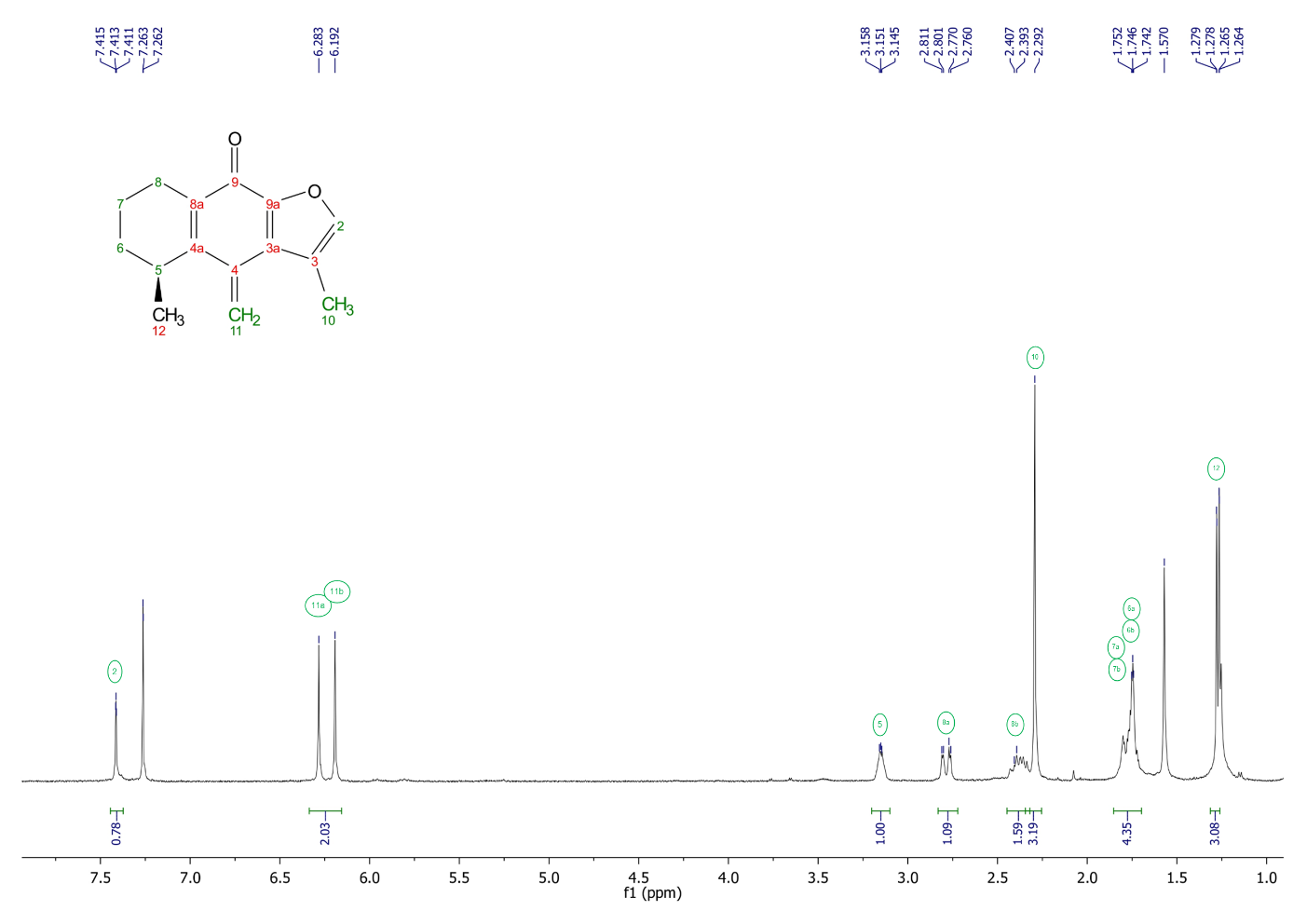
**Figure S4.** ^1^H NMR spectrum of methylenecyclohexadienone derivative (MTC) (**2**) (500 MHz, CDCl_3_).


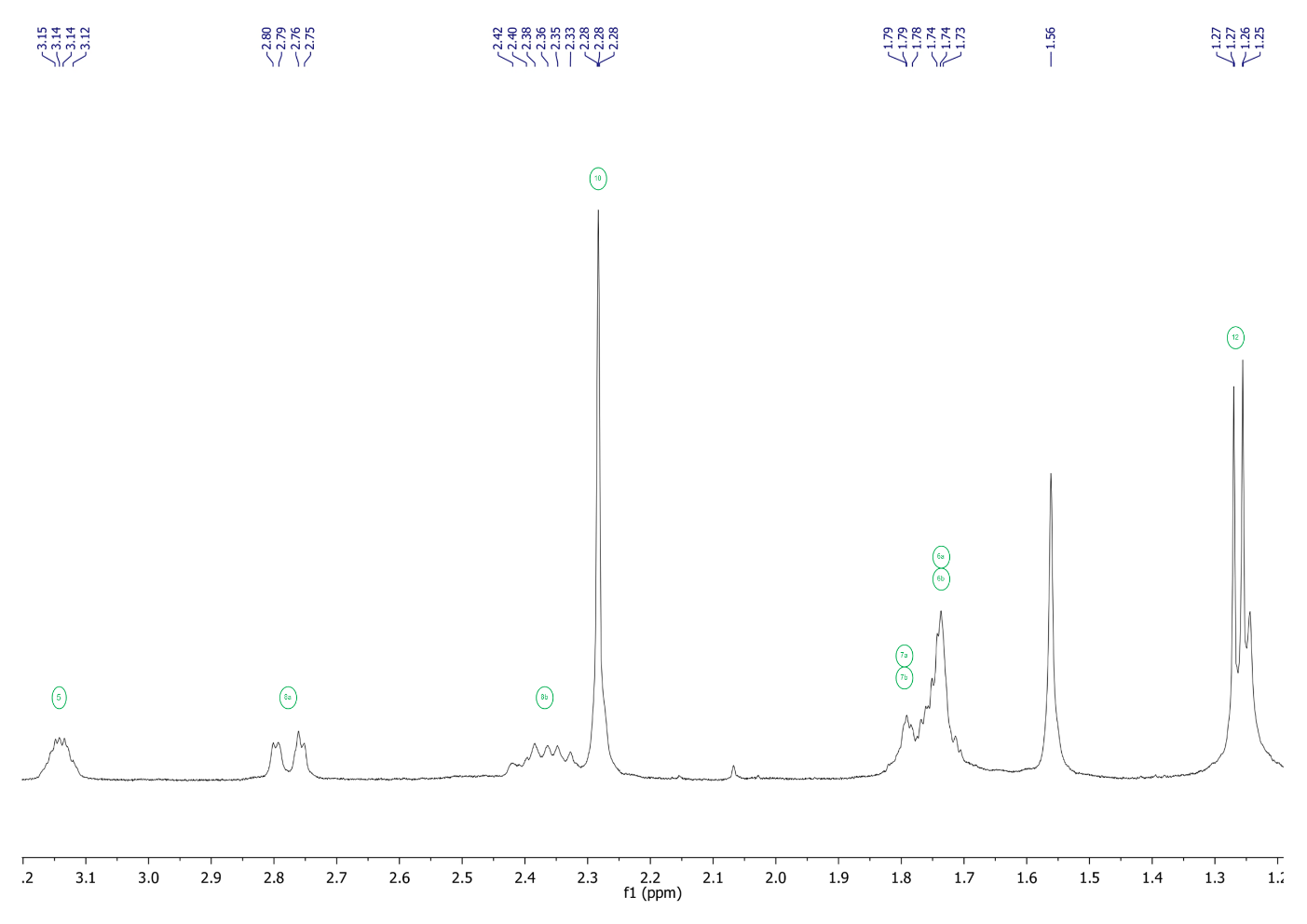


**Figure S4a.** Region of the ^1^H NMR spectrum of (*δ* 3.20–1.20 ppm) of methylenecyclohexadienone derivative (MTC) (**2**)

(500 MHz, CDCl_3_).


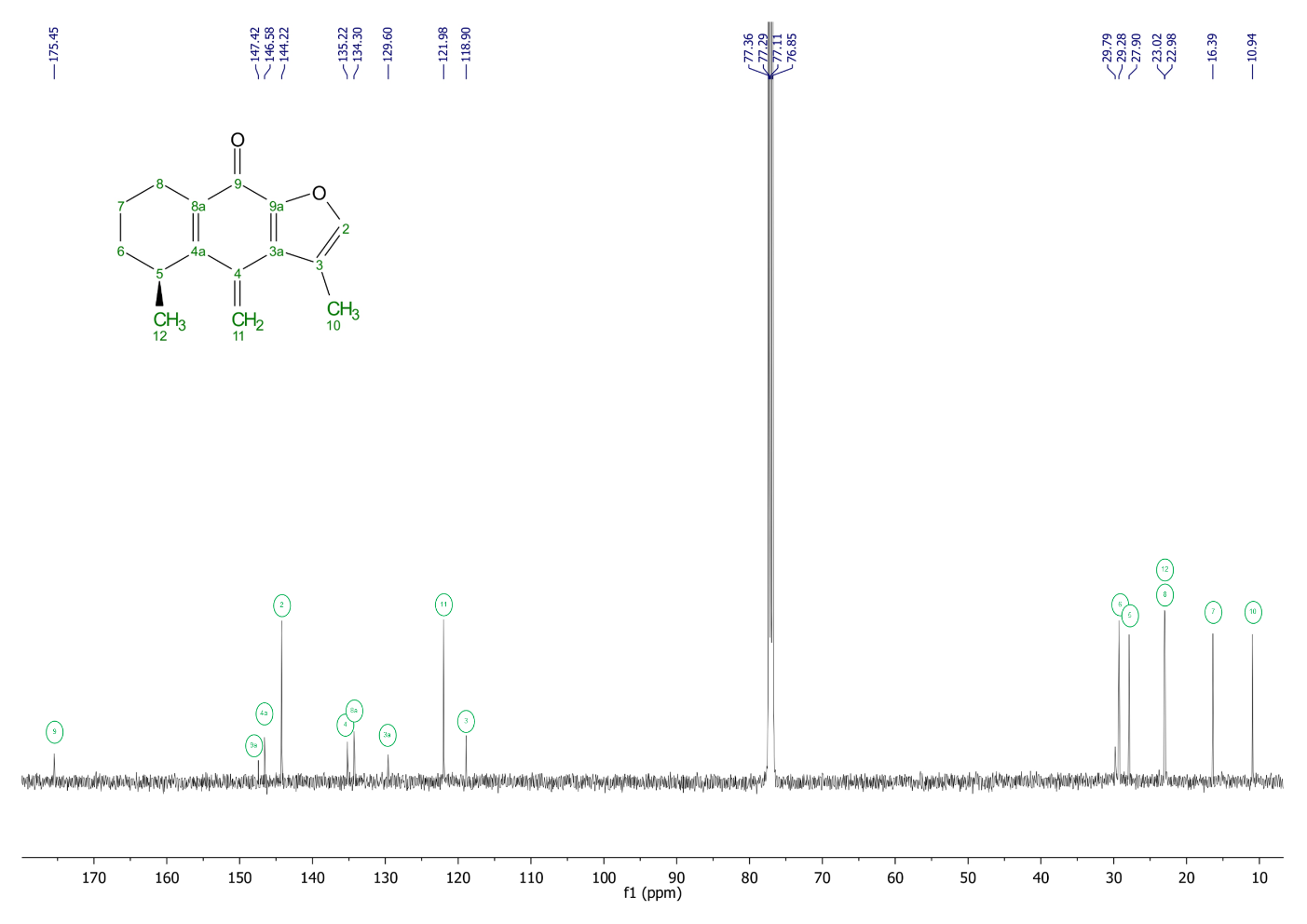


**Figure S5.** ^13^C NMR spectrum of methylenecyclohexadienone derivative (MTC) (**2**) (125.8 MHz, CDCl_3_).

**Figure S6.** HRESIMS of methylenecyclohexadienone derivative (MTC) (**2**).


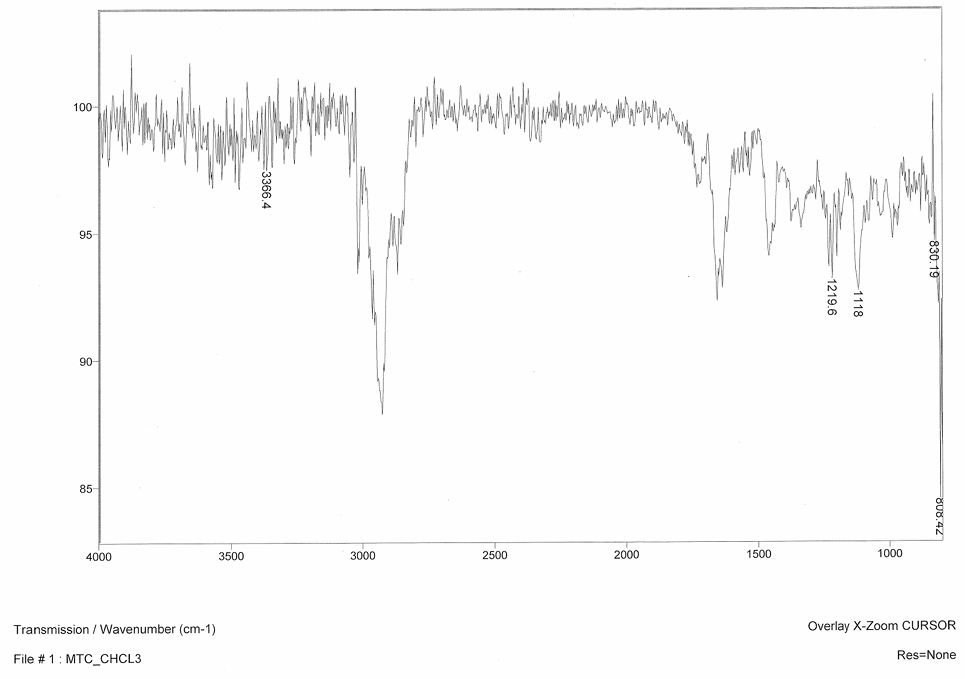


**Figure S7.** IR spectrum of methylenecyclohexadienone derivative (MTC) (**2**).

**
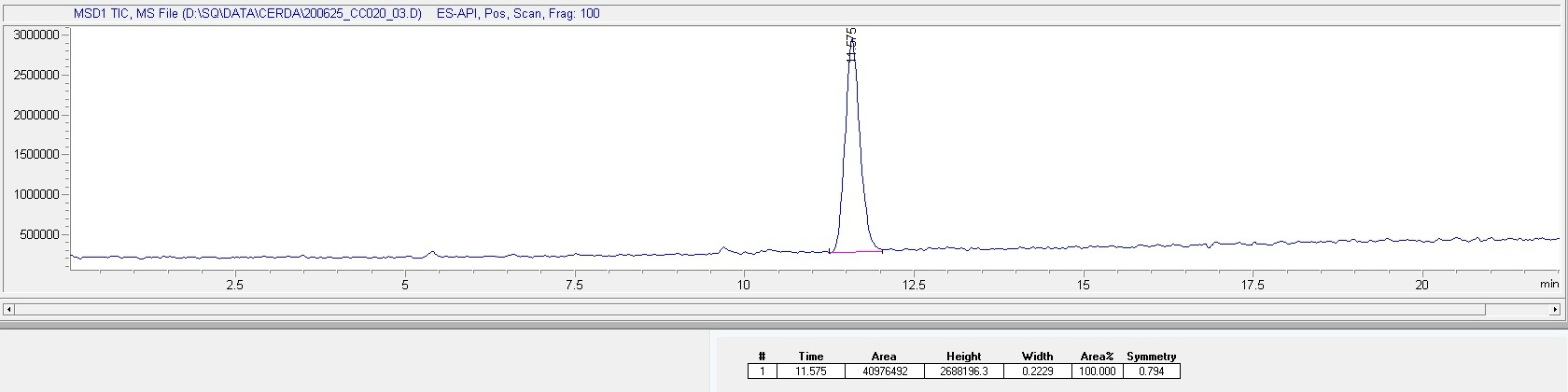
**

Peak results

| **#** | **Time (min)** | **Area** | **Height** | **Width** | **Area (%)** | **Symmetry** |
| --- | --- | --- | --- | --- | --- | --- |
| 1 | 11.575 | 40976492.0 | 2688196.3 | 0.2229 | 100.0 | 0.794 |

**Figure S8.** HPLC-MS of methylenecyclohexadienone derivative (MTC) (**2**).


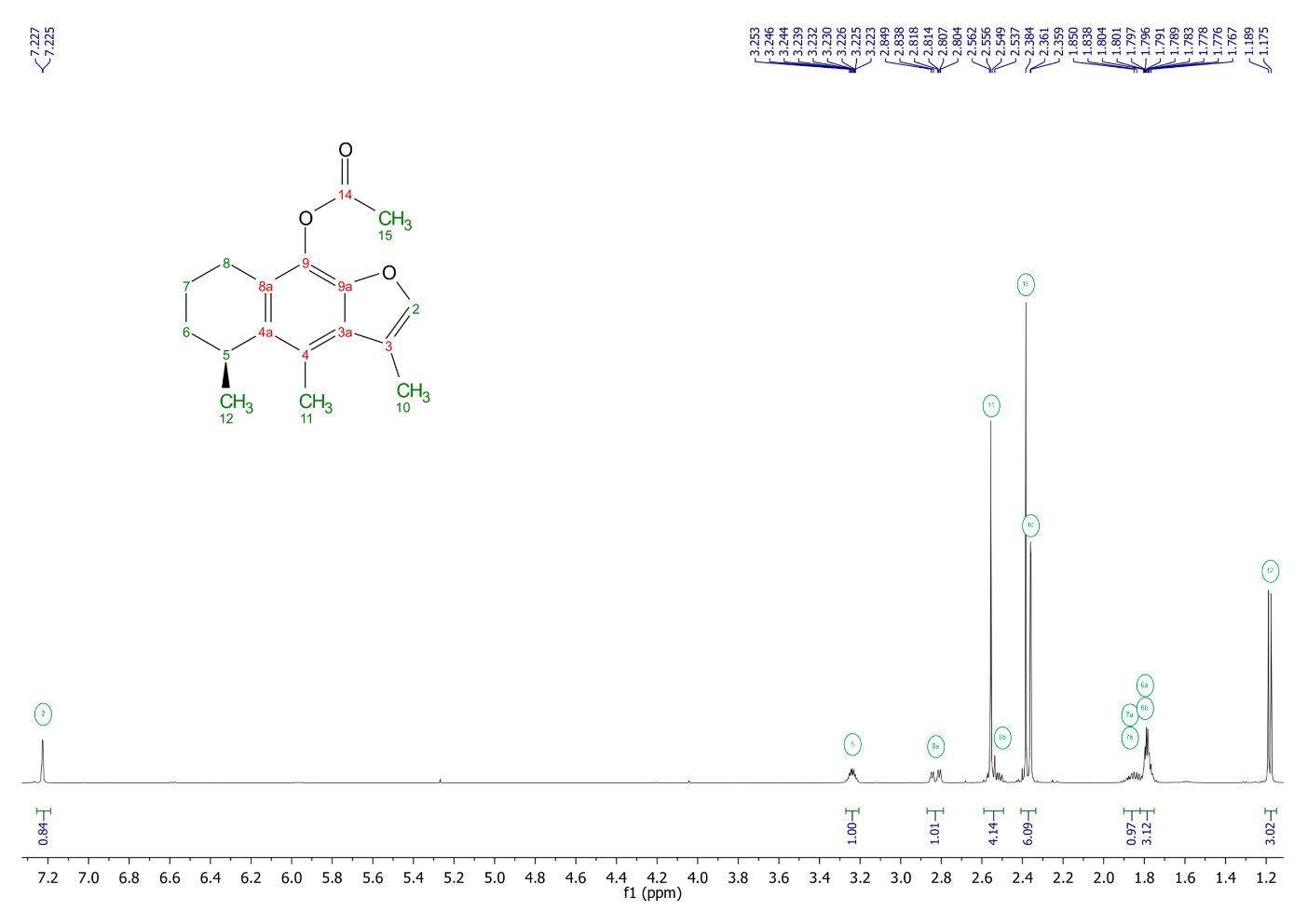


**Figure S9.** ^1^H NMR spectrum of cacalol acetate (**3**) (500 MHz, CDCl_3_).


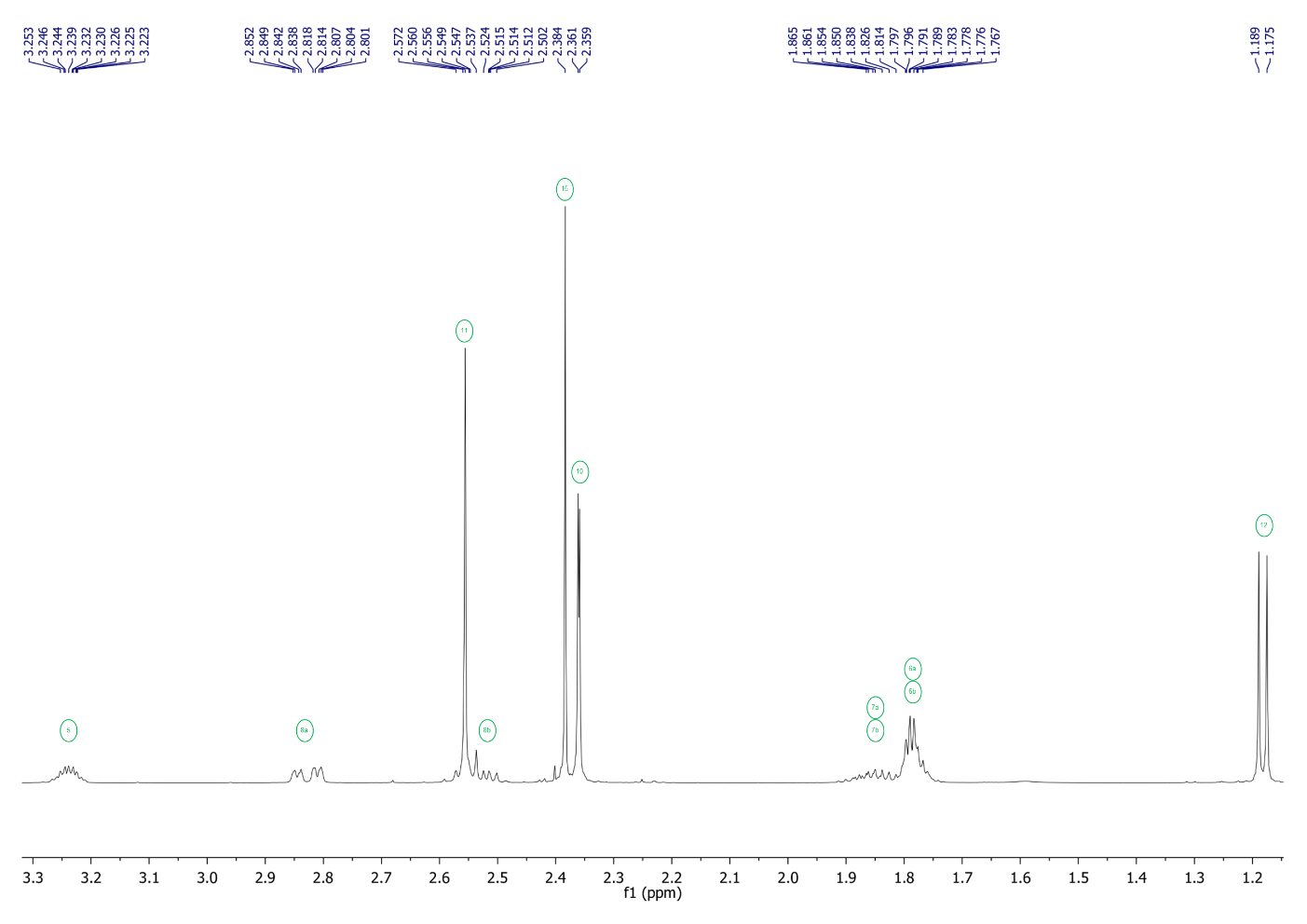


**Figure S9a.** Region of the ^1^H NMR spectrum (*δ* 3.30–1.15 ppm) of cacalol acetate (**3**) (500 MHz, CDCl_3_).


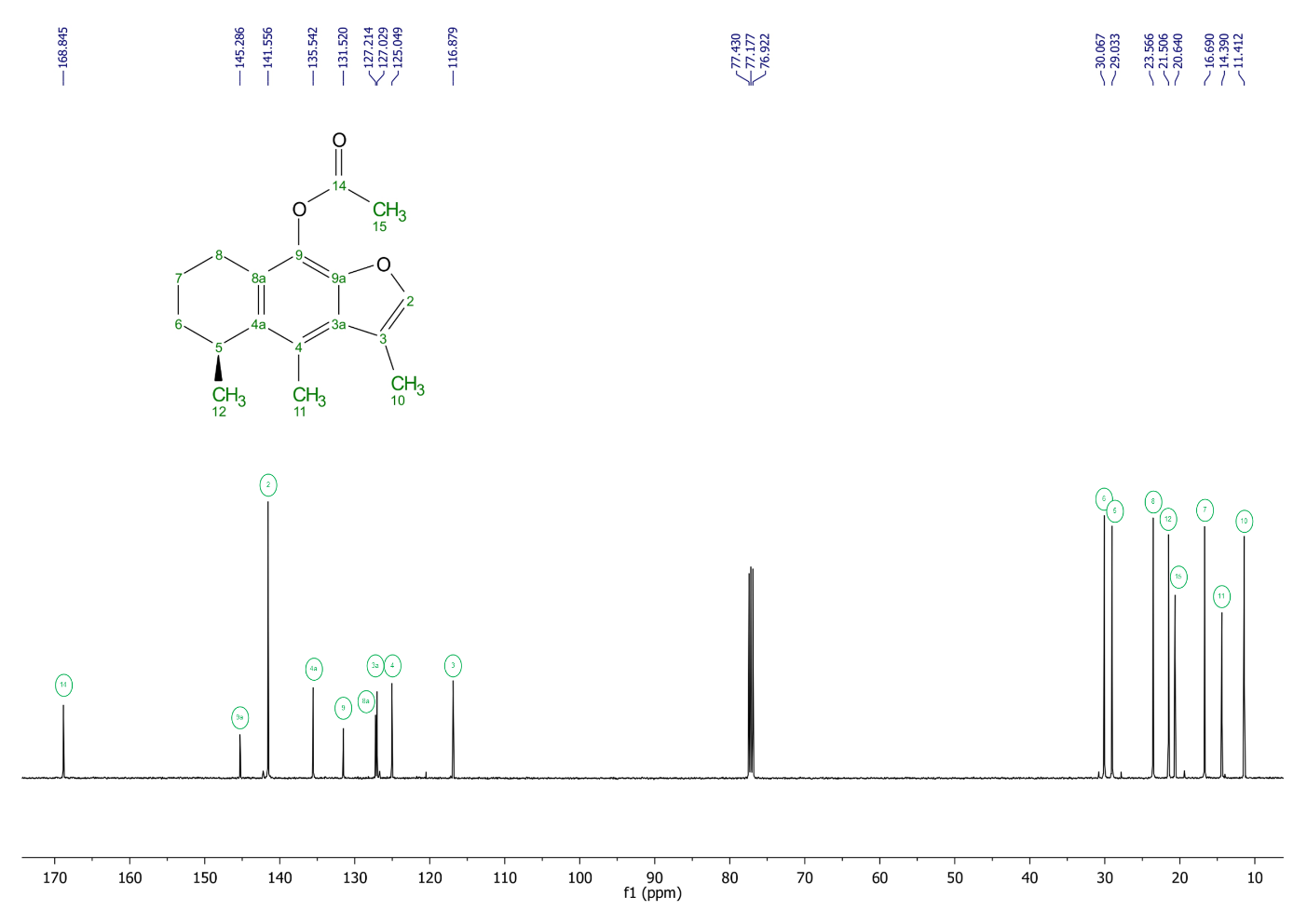


**Figure S10.** ^13^C NMR spectrum of cacalol acetate (**3**) (125.8 MHz, CDCl_3_).


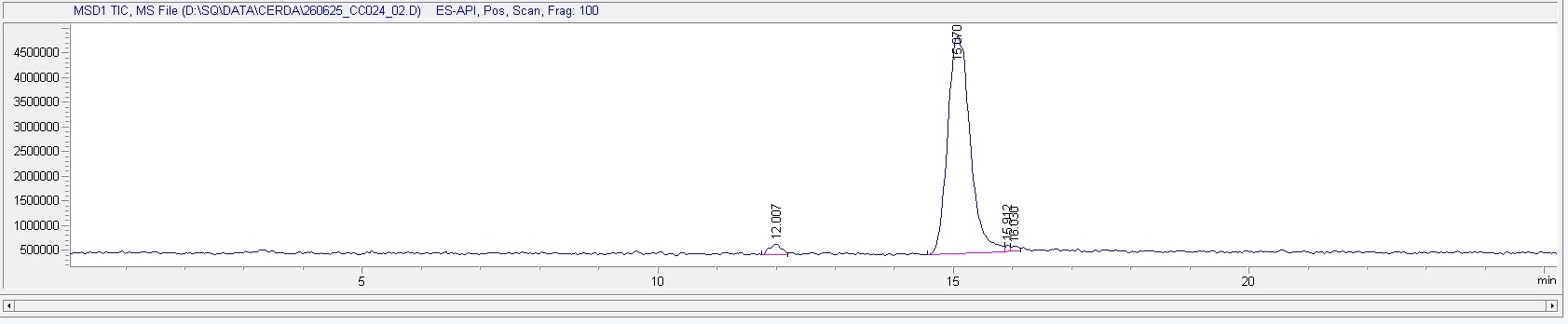


**Peak results**

| **#** | **Time (min)** | **Area** | **Height** | **Width** | **Area %** | **Symmetry** |
| --- | --- | --- | --- | --- | --- | --- |
| 1 | 12.007 | 3220487.3 | 218027.6 | 0.2061 | 2.647 | 1.368 |
| 2 | 15.070 | 116890456.0 | 4421247.0 | 0.3648 | 96.065 | 0.782 |
| 3 | 15.912 | 681365.2 | 133916.9 | 0.0706 | 0.560 | 0.908 |
| 4 | 16.030 | 885727.4 | 104651.6 | 0.1350 | 0.728 | 0.71 |

**Figure S11.** HPLC-MS of cacalol acetate (**3**).


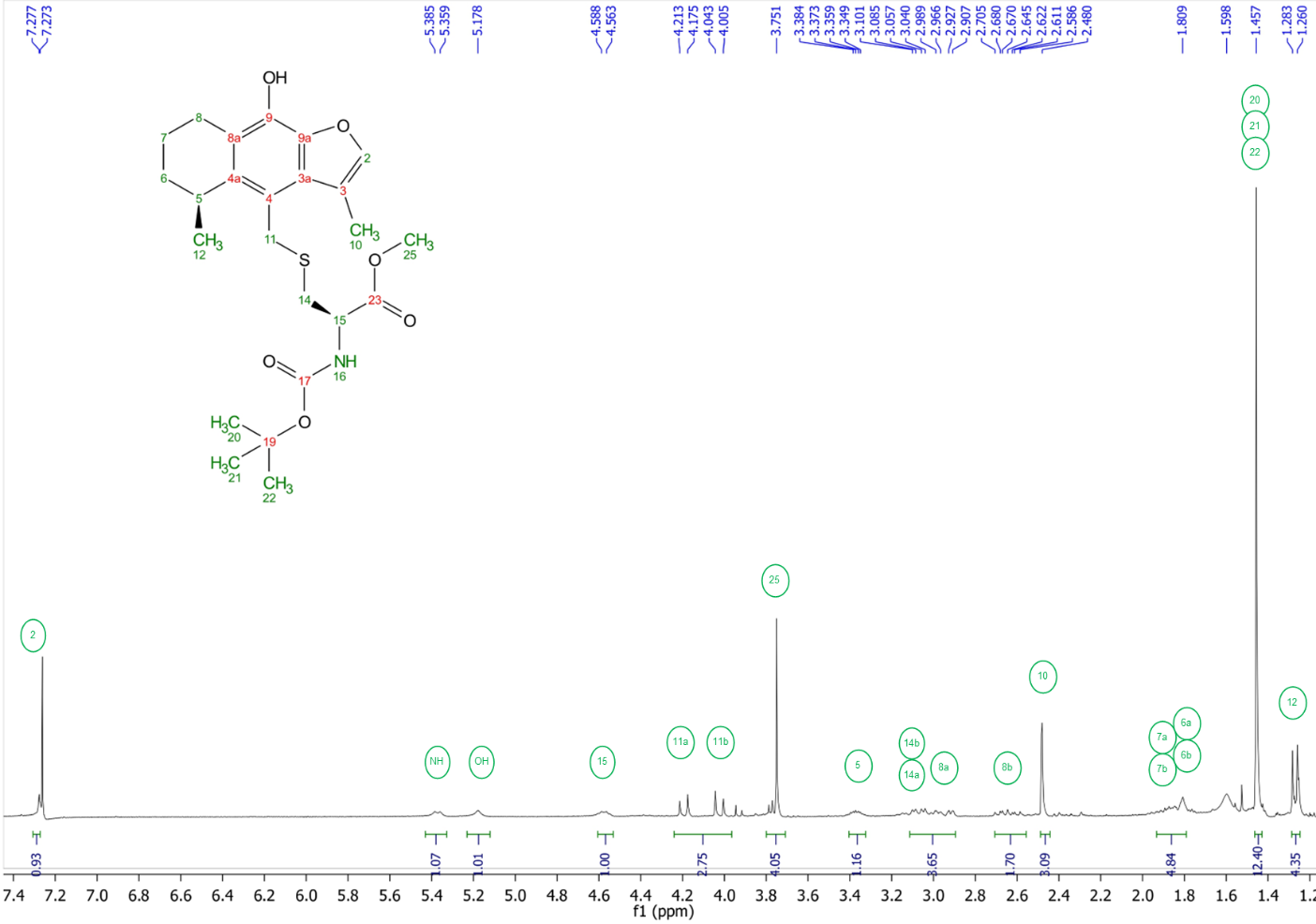


**Figure S12.** ^1^H NMR spectrum of compound **4** (300 MHz, CDCl_3_).


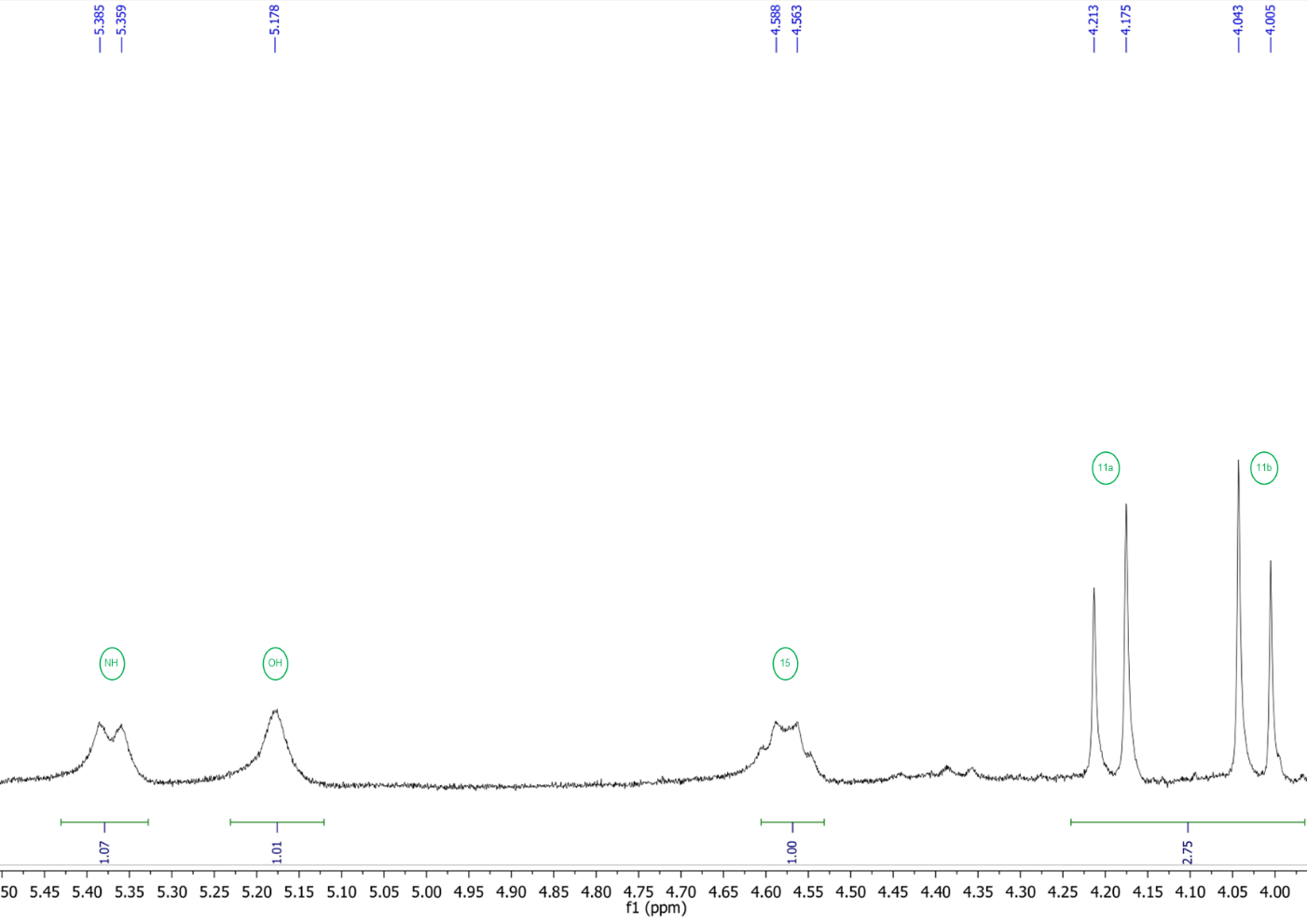


**Figure S12a.** Region of the ^1^H NMR spectrum (*δ* 5.50–3.97 ppm) of **4** (300 MHz, CDCl_3_).


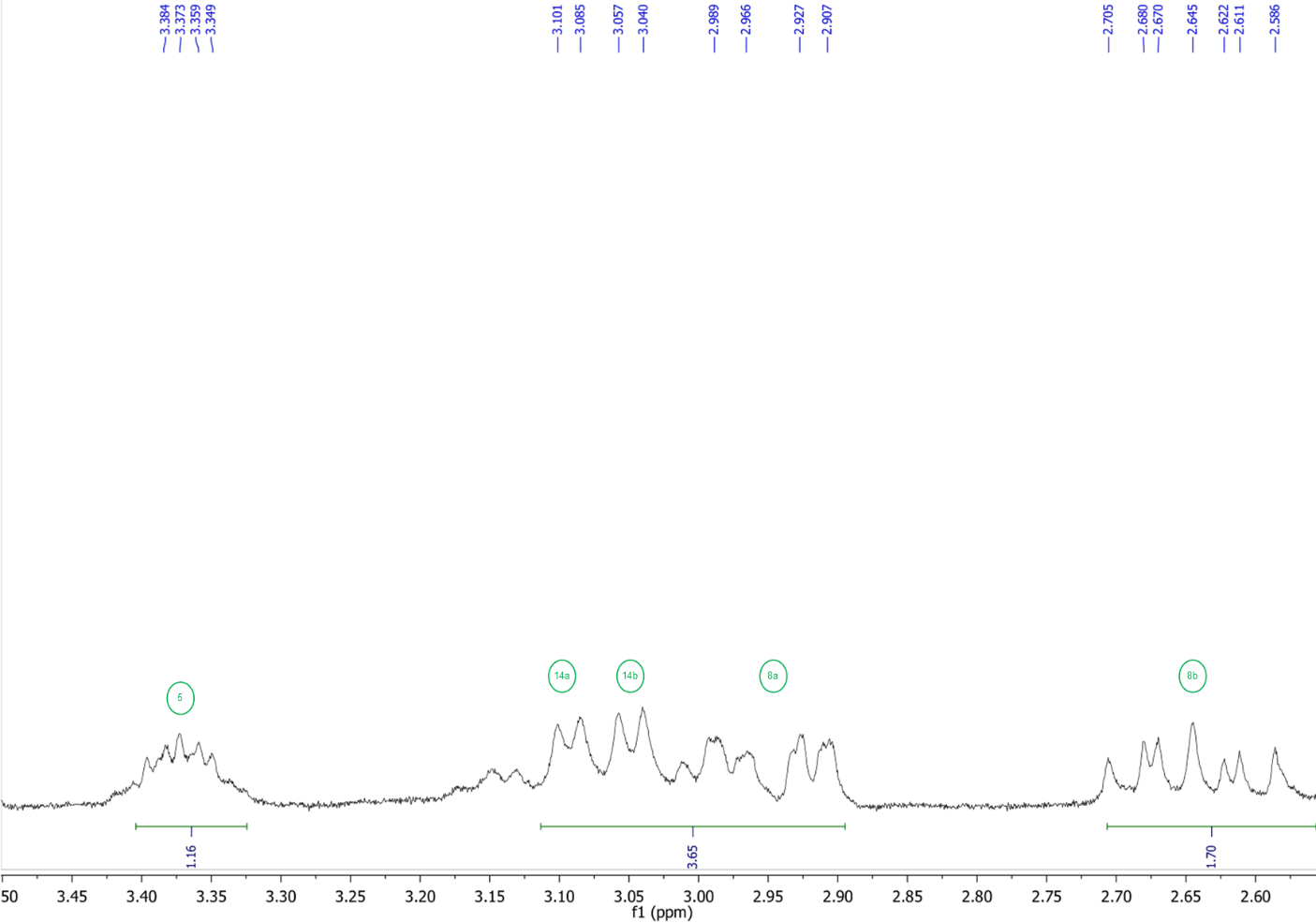


**Figure S12b.** Region of the ^1^H NMR spectrum (*δ* 3.50–2.56 ppm) of **4** (300 MHz, CDCl_3_).


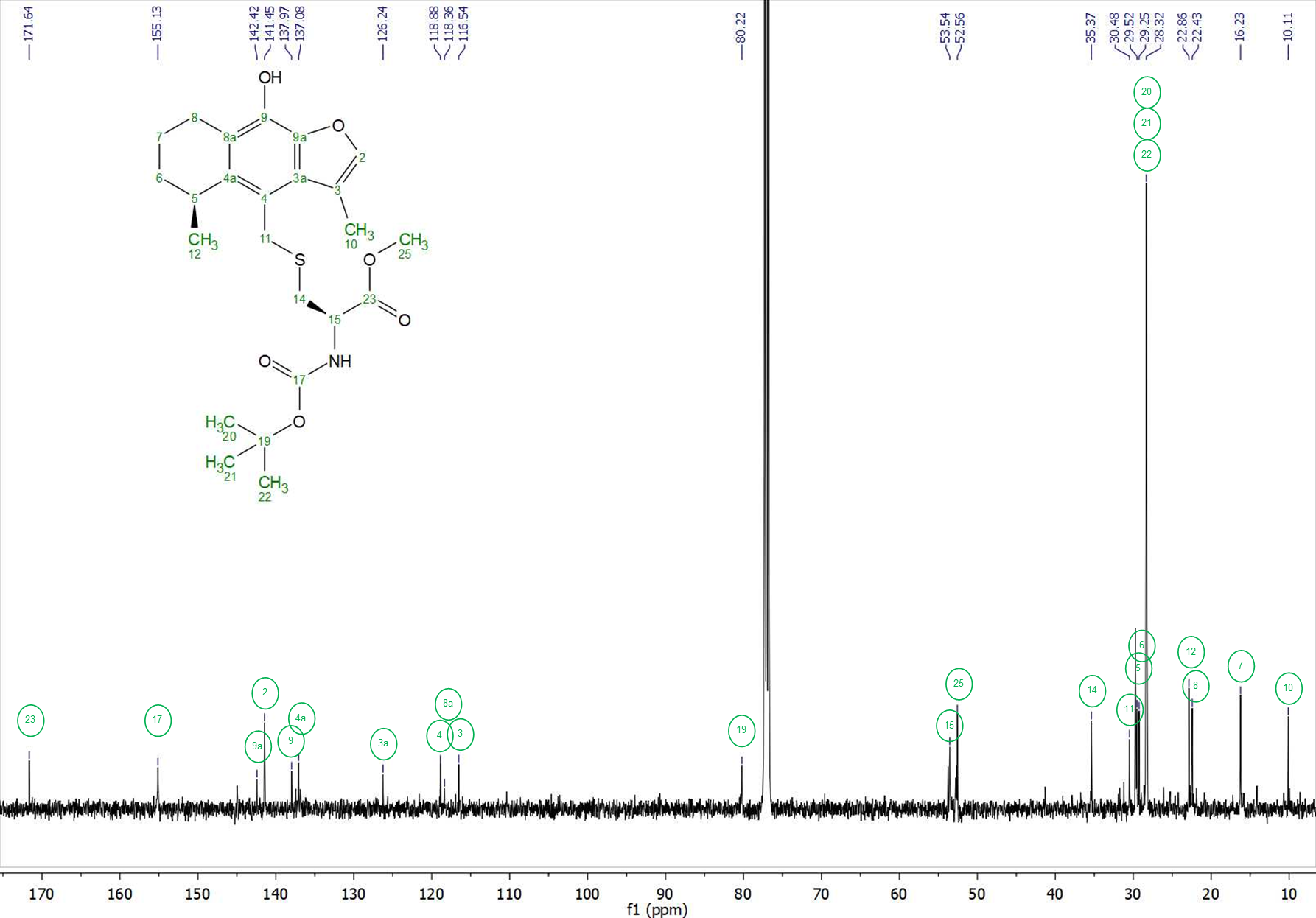


**Figure S13.** ^13^C NMR spectrum of compound of **4** (125.8 MHz, CDCl_3_).


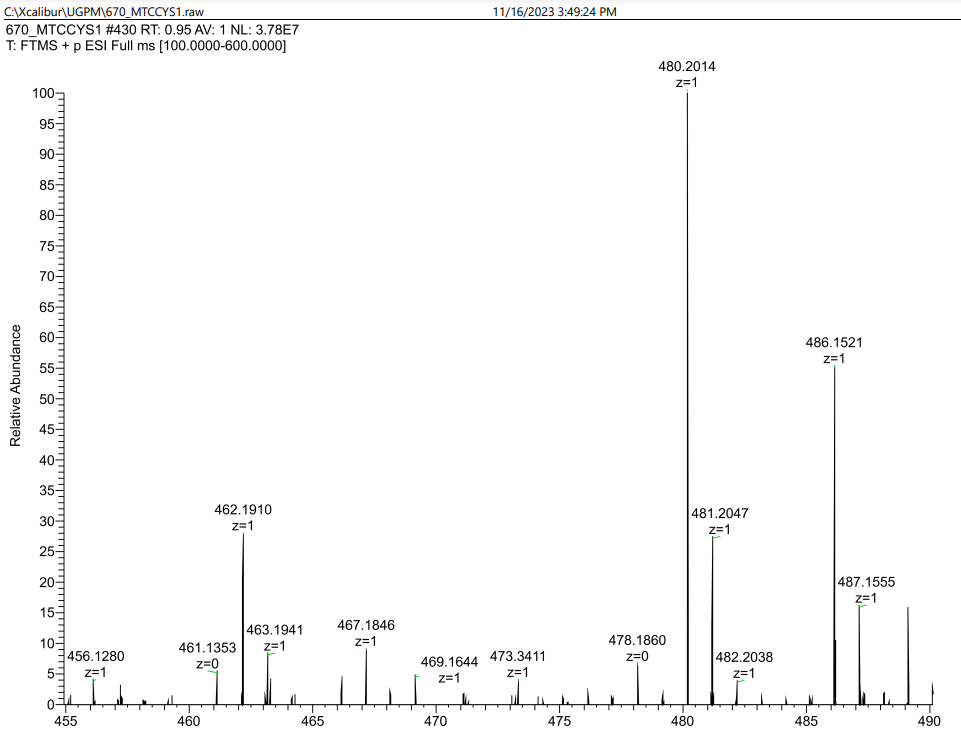


**Figure S14.** HRESIMS of **4**.


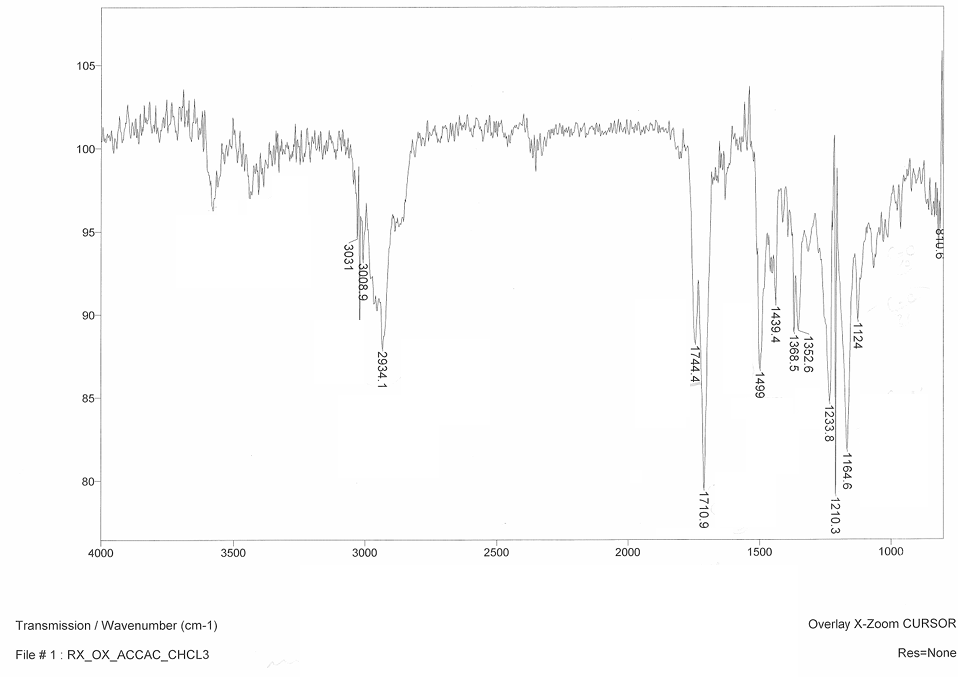


**Figure S15.** IR spectrum of **4**.


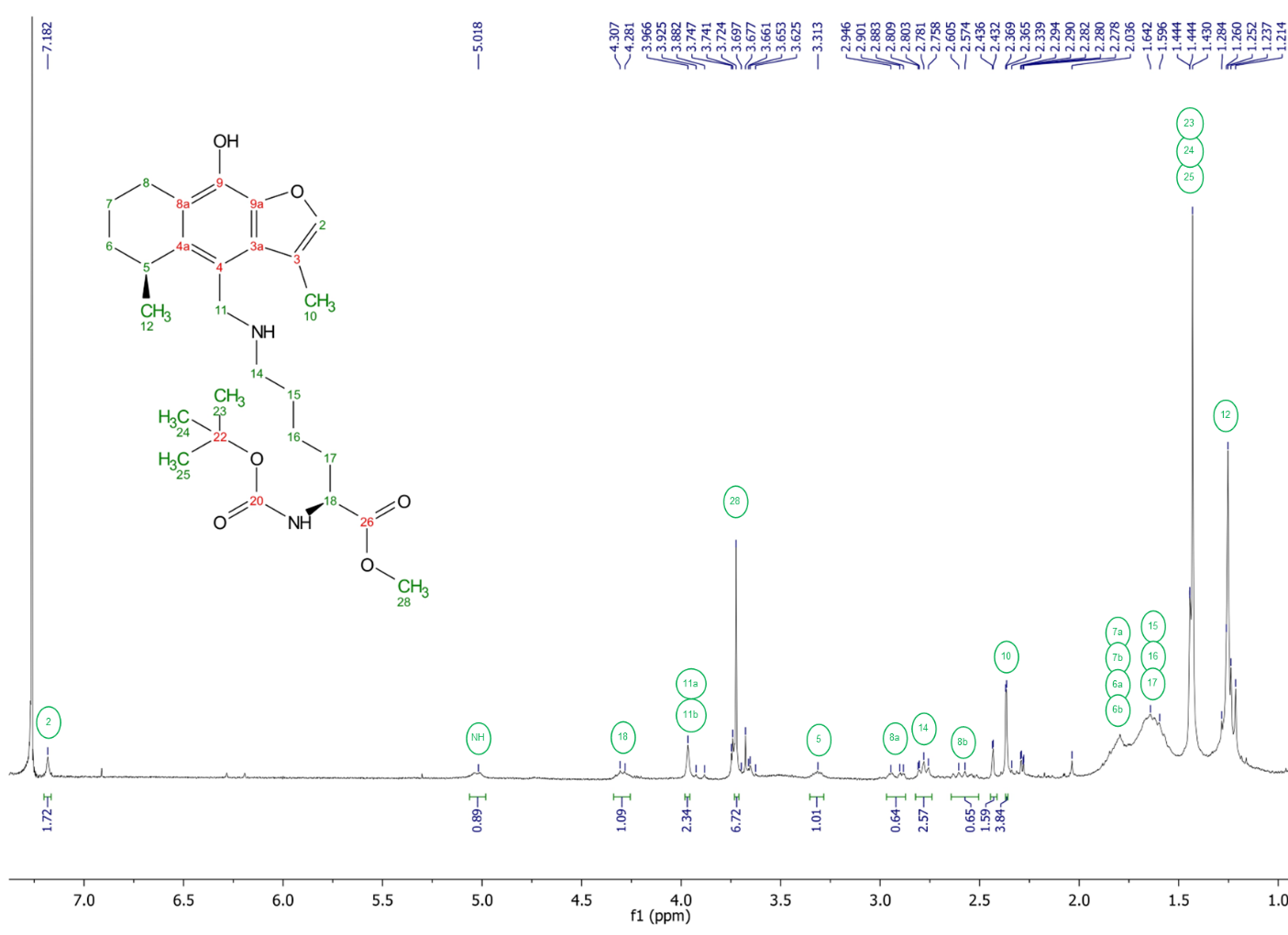


**Figure S16.** ^1^H NMR spectrum of compound **5** (300 MHz, CDCl_3_).


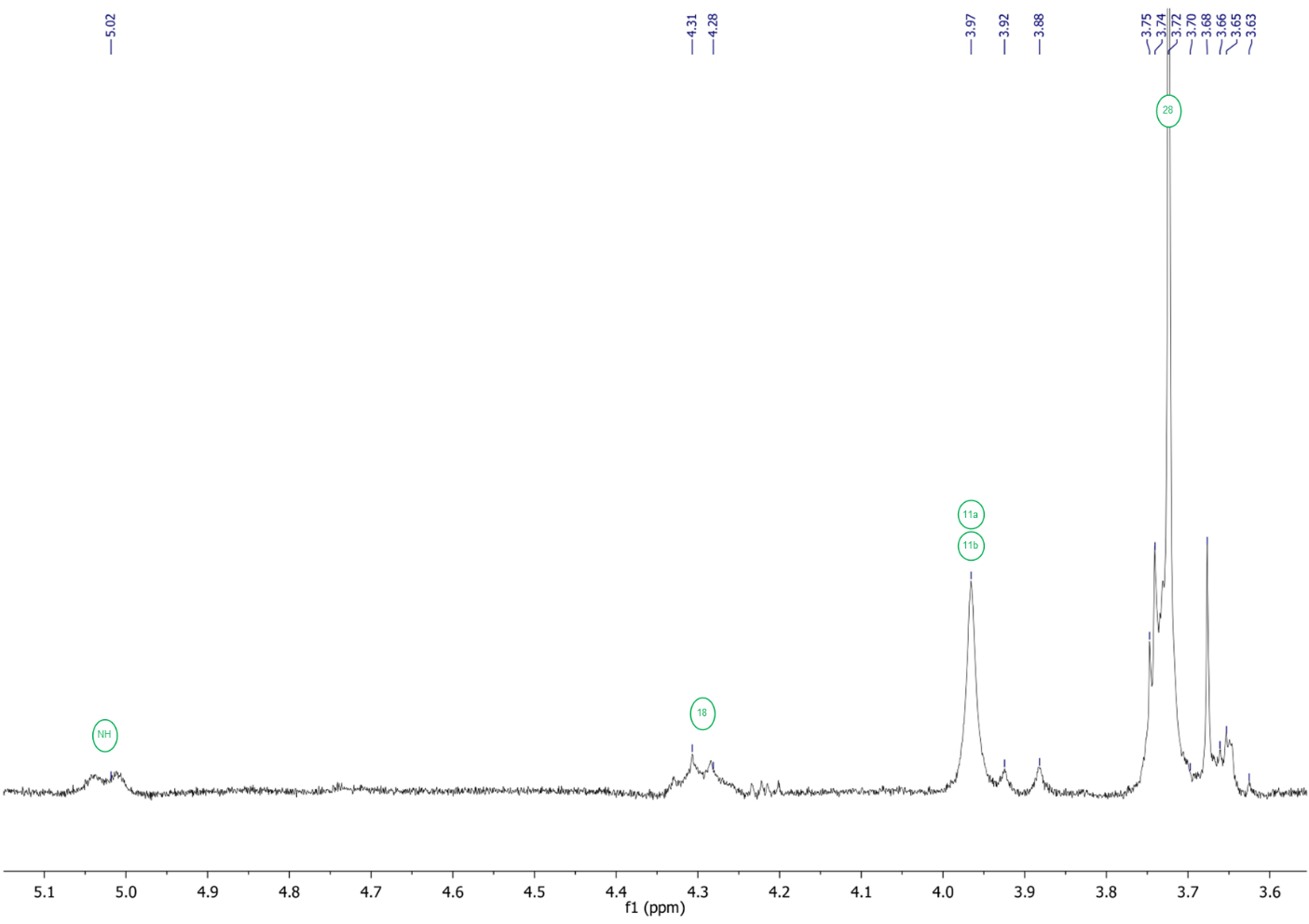


**Figure S16a.** Region of the ^1^H NMR spectrum (*δ* 5.14–3.56 ppm) of **5** (300 MHz, CDCl_3_).


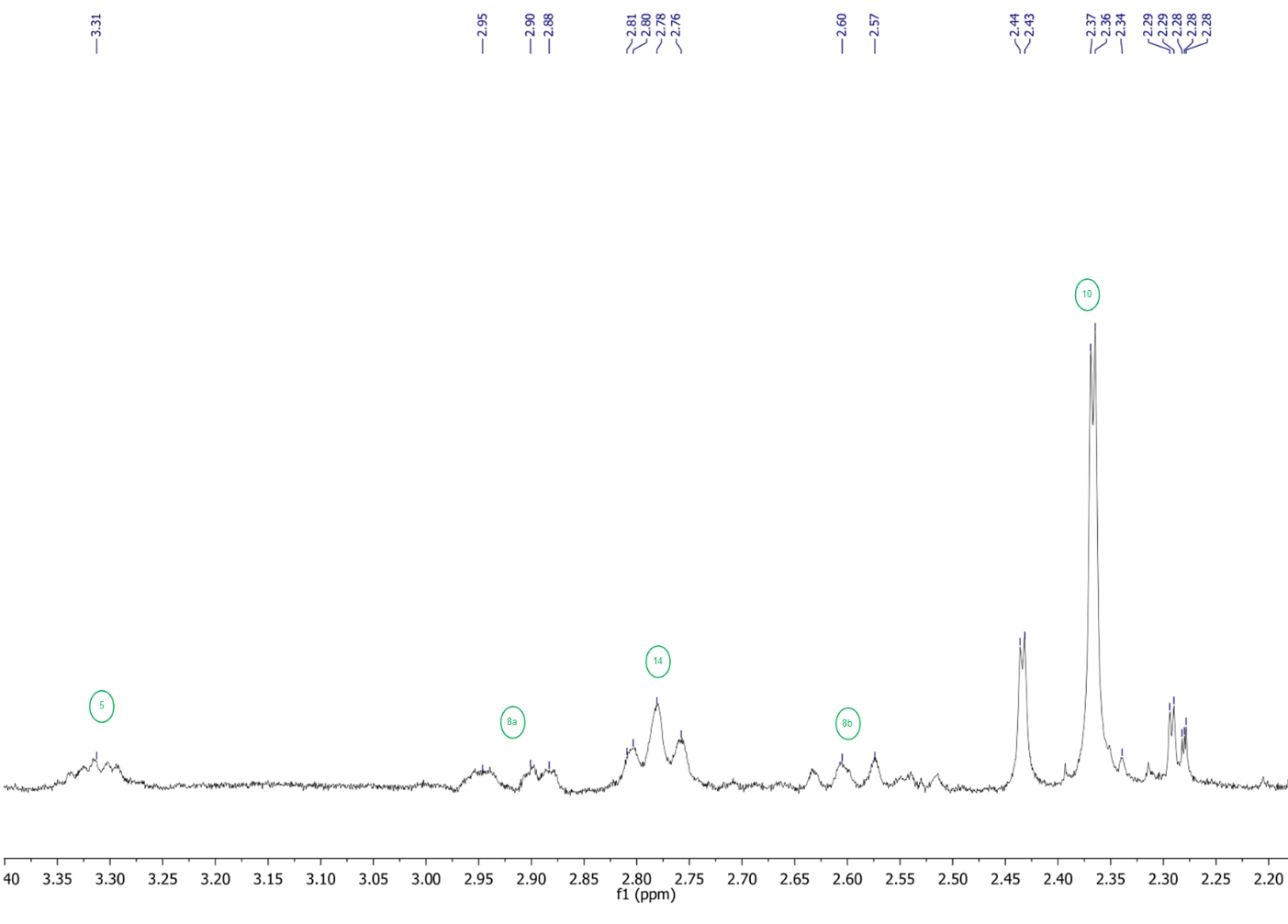


**Figure S16b.** Region of the ^1^H NMR spectrum (*δ* 3.40–2.20 ppm) of **5** (300 MHz, CDCl_3_).


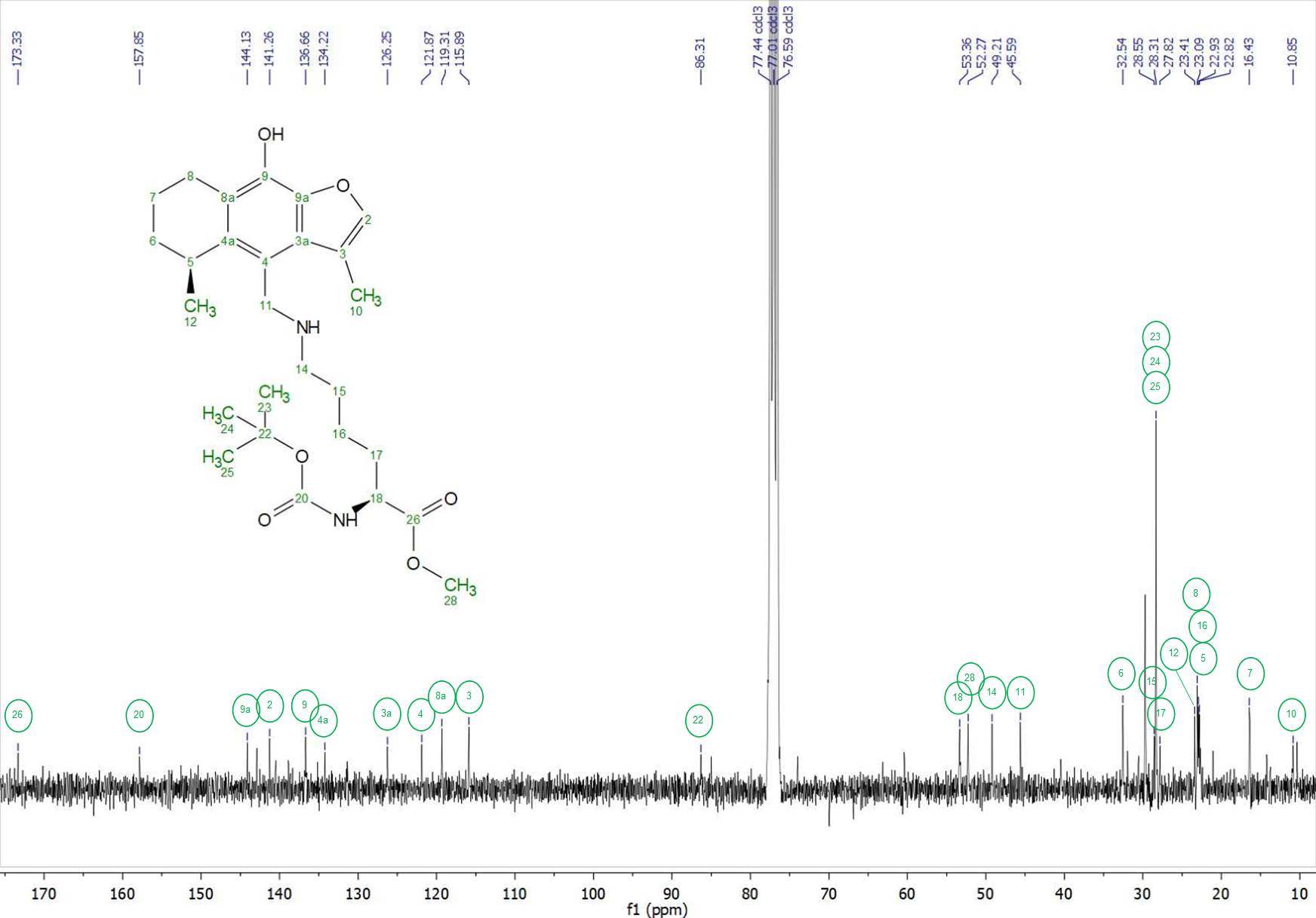


**Figure S17.** ^13^C NMR spectrum of compound **5** (75.4 MHz, CDCl_3_).


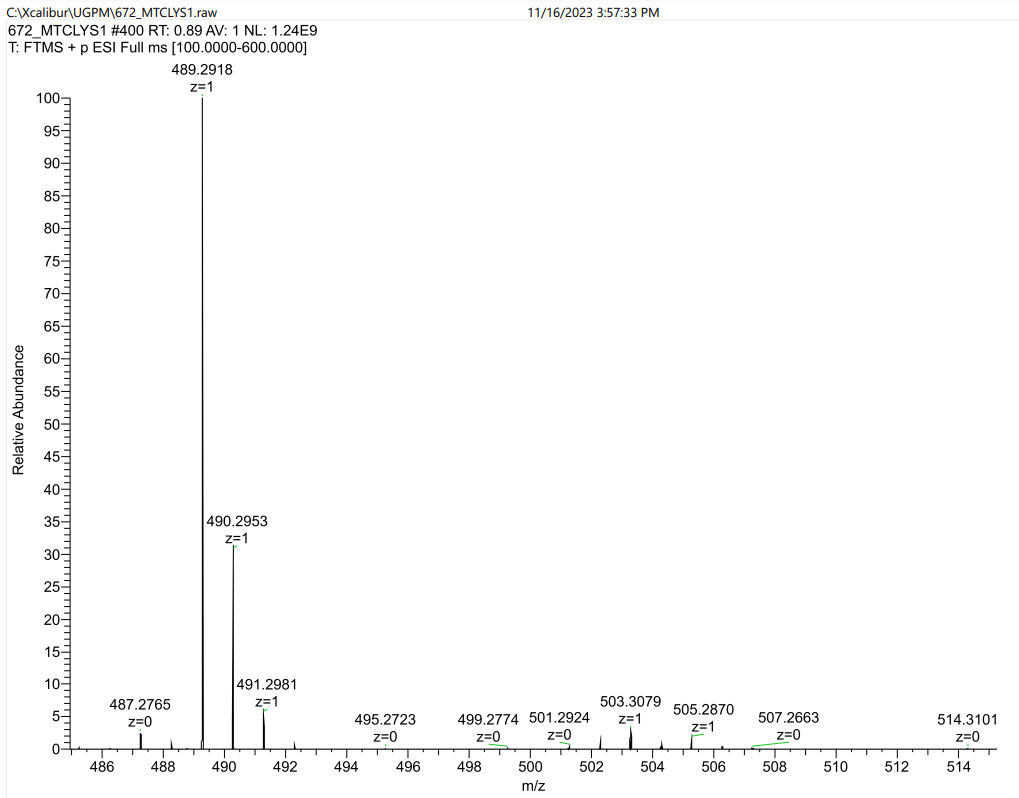


**Figure S18.** HRESIMS of **5**.


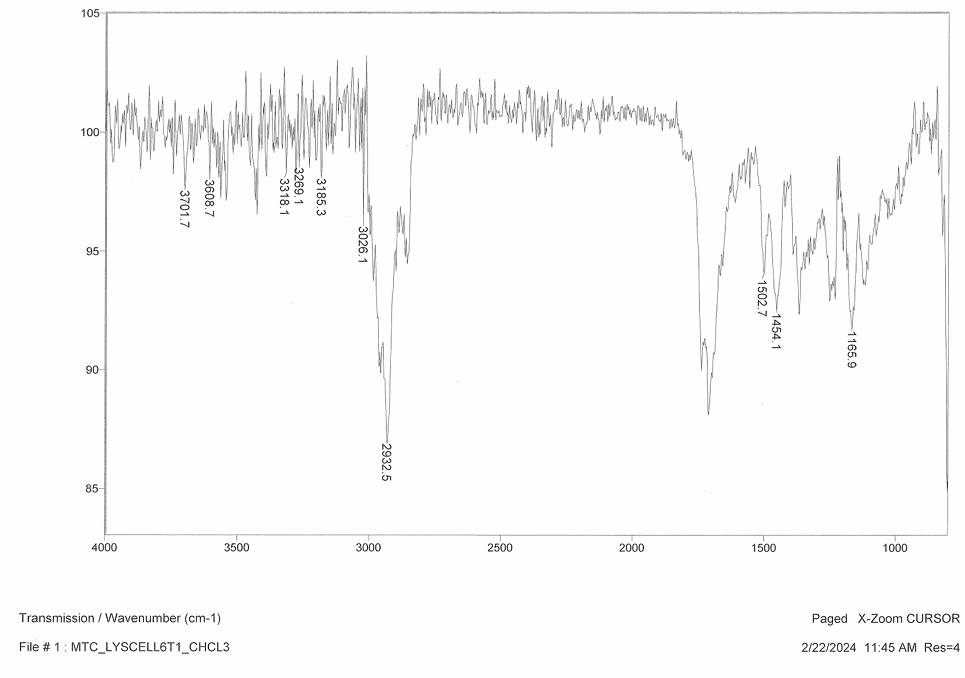


**Figure S19.** IR spectrum of **5**.


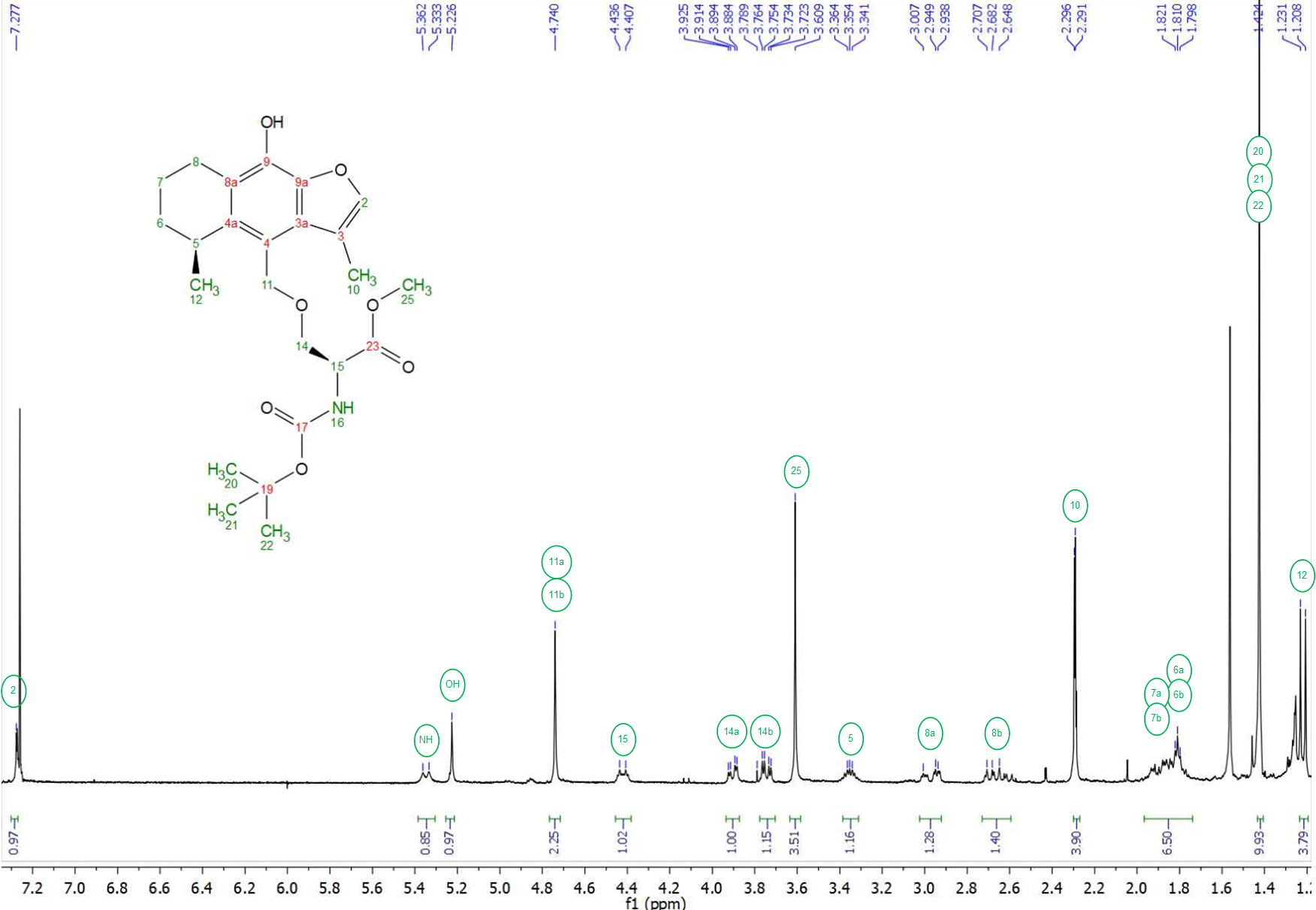


**Figure S20.** ^1^H NMR spectrum of compound **6** (300 MHz, CDCl_3_).


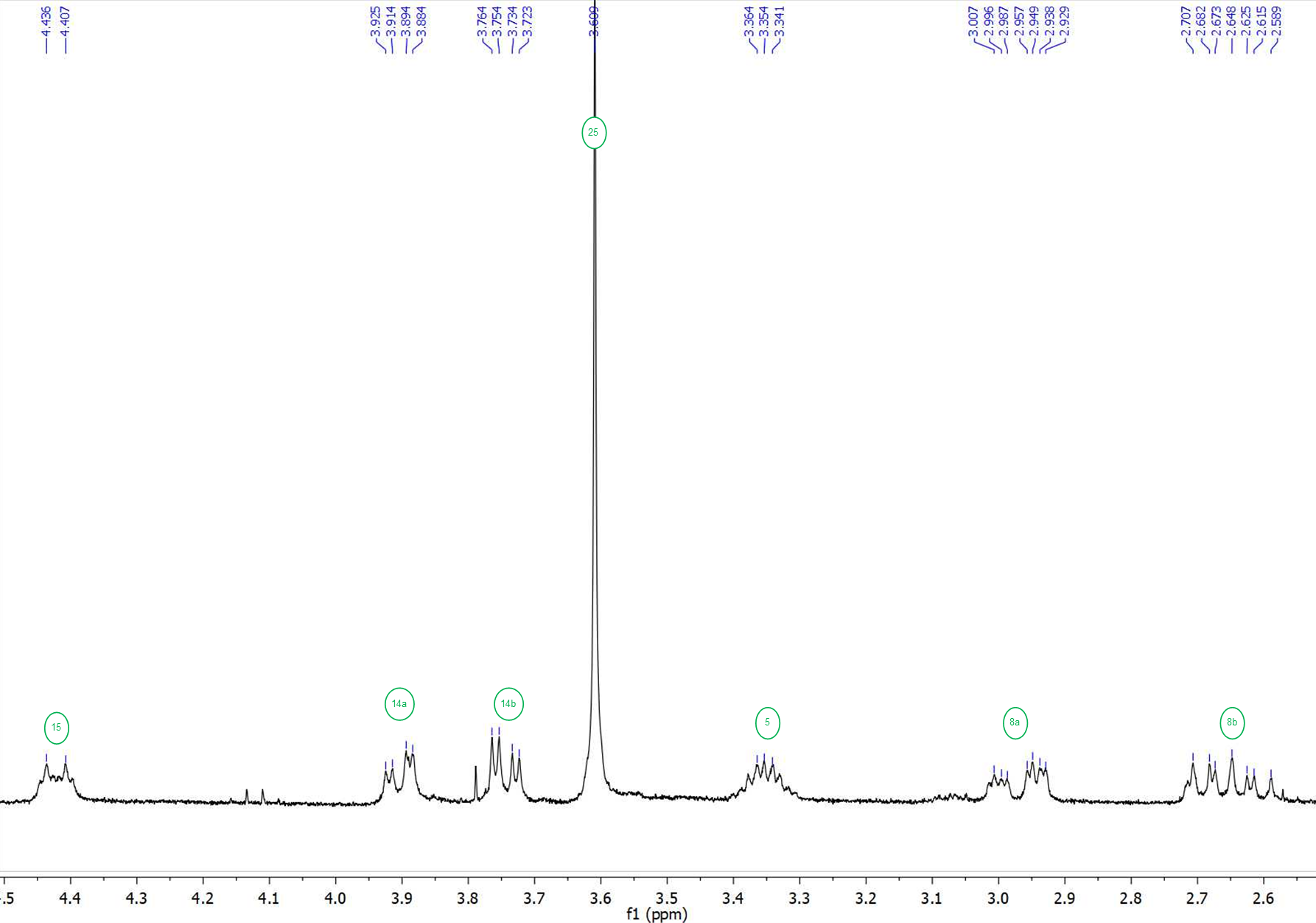


**Figure S20a.** Region of the ^1^H NMR spectrum (*δ* 4.50–2.52 ppm) of **6** (300 MHz, CDCl_3_).


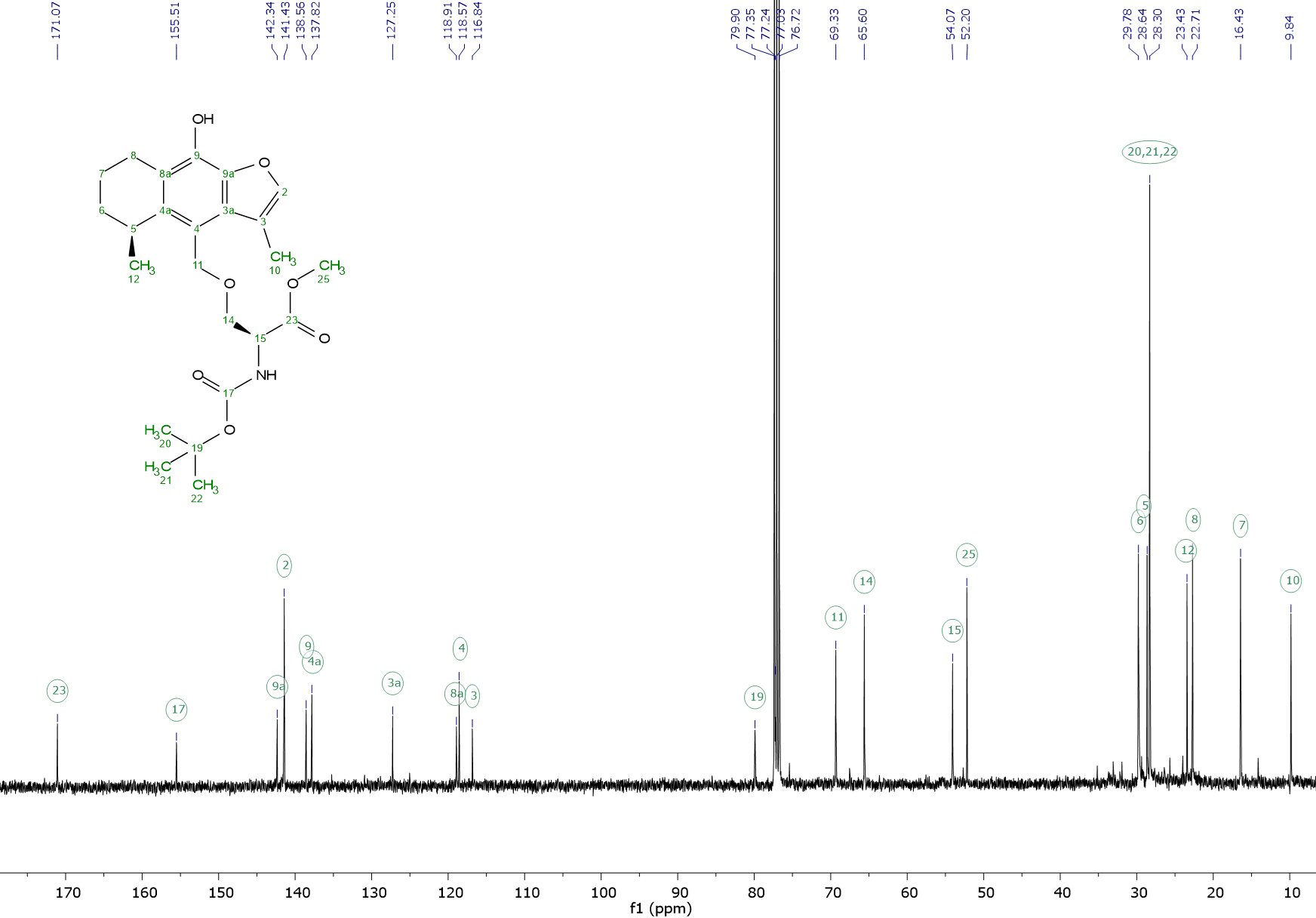


**Figure S21.** ^13^C NMR spectrum of compound **6** (100 MHz, CDCl_3_).

**
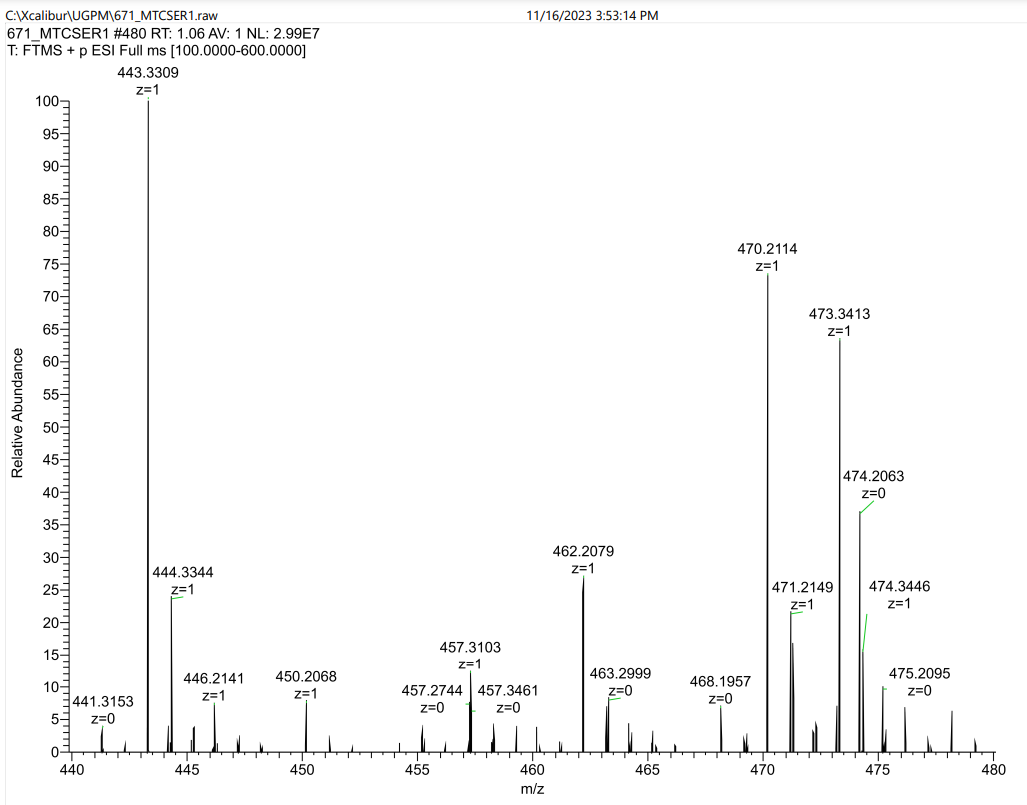
**

**Figure S22.** HRESIMS of **6**.


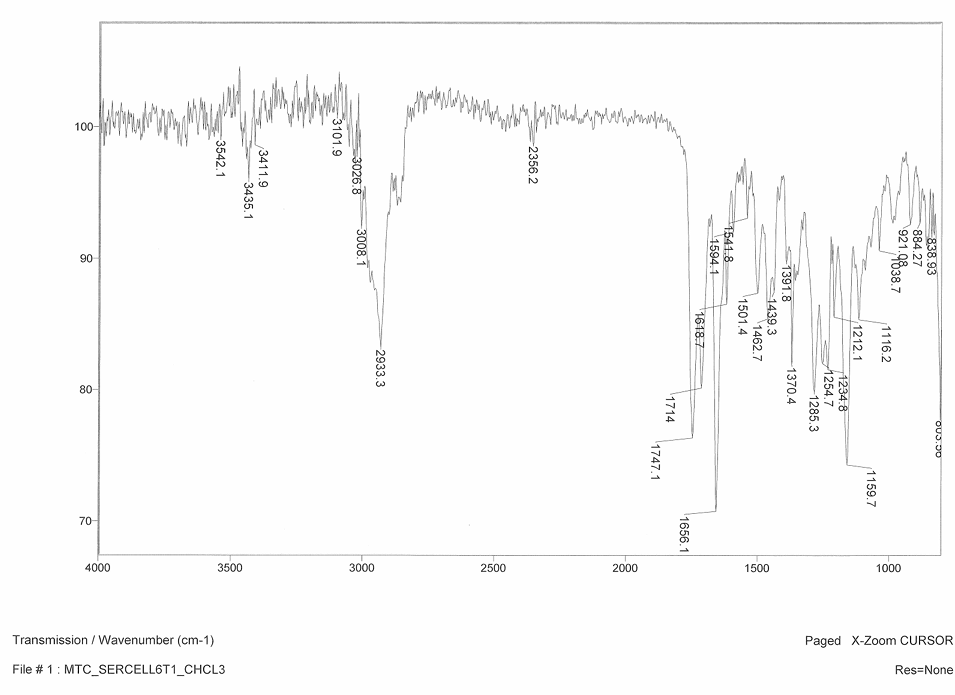


**Figure S23.** IR spectrum of **6**.


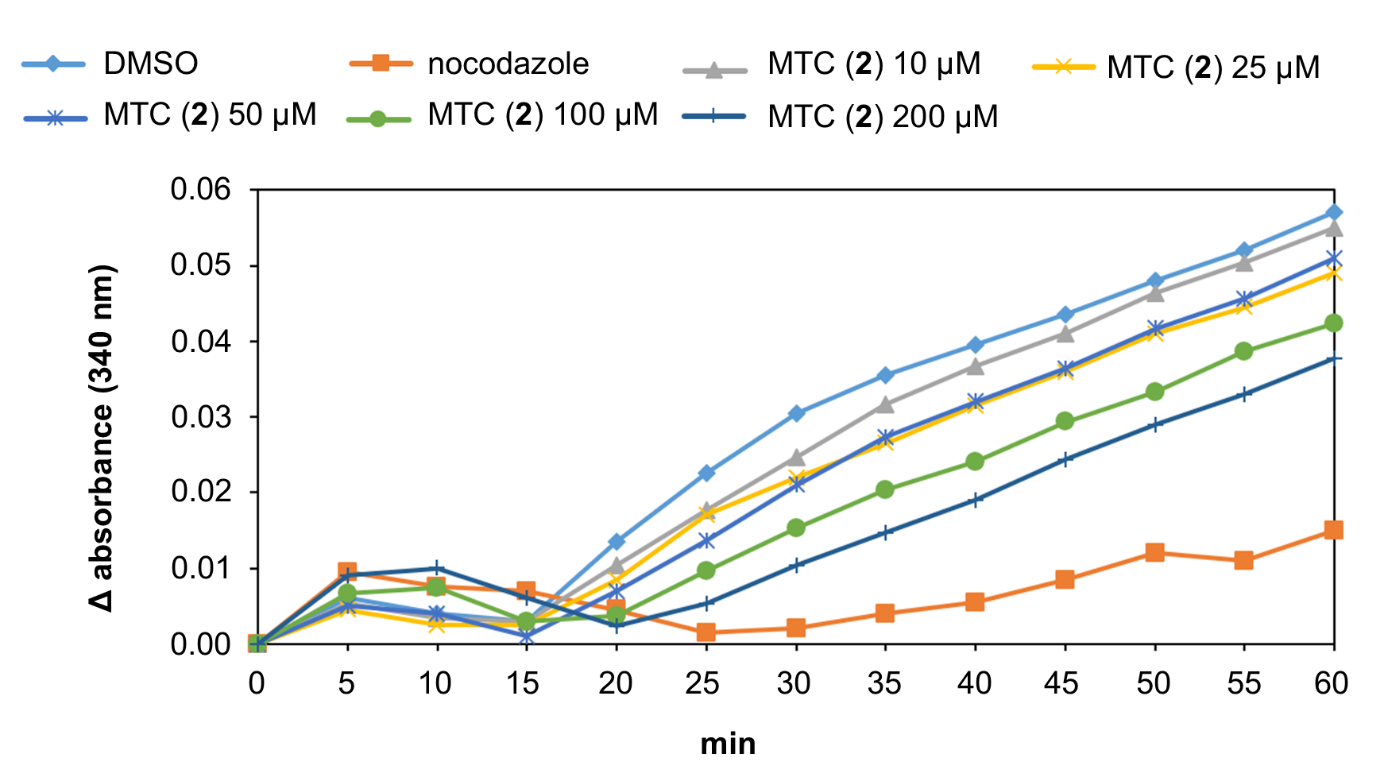


**Figure S24.** Tubulin polymerization curves of MTC (**2**).


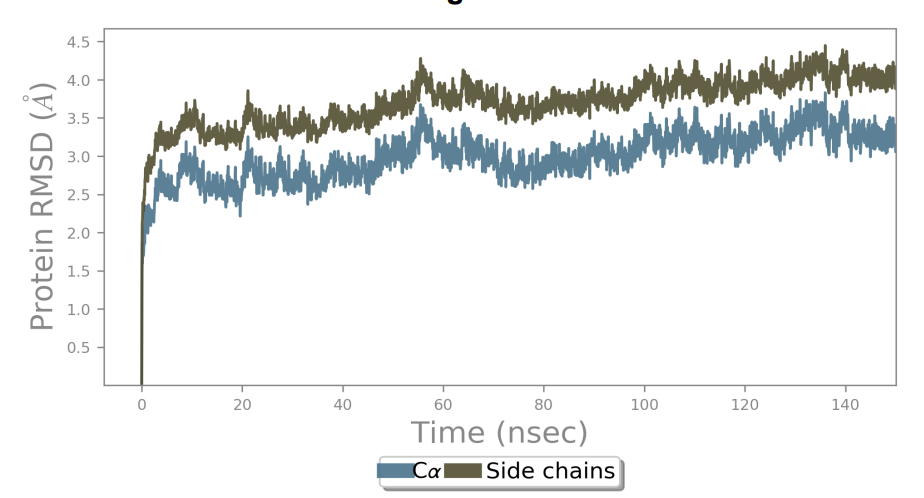


**Figure S25.** Molecular dynamics results of the complex α-tubulin-MTC.

**Appendix S1.** Yasara output for global docking result analysis

==============================

25 VINA docking runs of the ligand object 3 to the receptor object 1 yielded the following results, sorted by binding energy [more positive energies indicate stronger binding, and negative energies mean no binding] "Con.Surf" is the molecular contact surface.

Run |Bind.energy[kcal/mol]|Dissoc. constant [pM]| Con.Surf[A^2] | Contacting receptor residues

----+---------------------+---------------------+---------------+-----------------------------

001 | 000006.8420 | 00000009654894.0000 | 000204.45 | A ASP 306 A HIS 309 A GLY 310 A THR 382 A ALA 383 A ALA 385 A GLU 386 A TRP 388 A ALA 389 A GLU 429 A LYS 430 A TYR 432 A GLU 433 A VAL 437

002 | 000006.7250 | 00000011762771.0000 | 000192.12 | A ASP 306 A HIS 309 A GLY 310 A THR 382 A ALA 383 A ALA 385 A GLU 386 A ALA 389 A GLU 429 A TYR 432 A GLU 433 A VAL 437

003 | 000006.6280 | 00000013855161.0000 | 000200.84 | A HIS 309 A GLY 310 A THR 382 A ALA 383 A ALA 385 A GLU 386 A ALA 389 A GLU 429 A LYS 430 A TYR 432 A GLU 433 A VAL 437

004 | 000006.3980 | 00000020426964.0000 | 000200.24 | A SER 158 A GLY 162 A LYS 163 A LYS 164 A SER 165 A LYS 166 A GLU 196 A HIS 197 A SER 198 A ASP 199 A GLN 256 A VAL 260 A PRO 263

005 | 000006.2320 | 00000027032326.0000 | 000183.92 | A ASP 306 A HIS 309 A GLY 310 A THR 382 A ALA 383 A ALA 385 A GLU 386 A ALA 389 A GLU 429 A TYR 432 A GLU 433 A VAL 437

006 | 000006.1630 | 00000030371140.0000 | 000198.94 | A SER 158 A GLY 162 A LYS 163 A LYS 164 A SER 165 A LYS 166 A GLU 196 A HIS 197 A SER 198 A ASP 199 A GLN 256 A VAL 260 A PRO 263

007 | 000005.9940 | 00000040396128.0000 | 000180.59 | A SER 158 A GLY 162 A LYS 163 A LYS 164 A SER 165 A LYS 166 A GLU 196 A HIS 197 A SER 198 A ASP 199 A GLN 256 A PRO 263

008 | 000005.9650 | 00000042422576.0000 | 000211.53 | A HIS 309 A GLY 310 A THR 382 A ALA 383 A ALA 385 A GLU 386 A TRP 388 A ALA 389 A GLU 429 A LYS 430 A TYR 432 A GLU 433 A VAL 437

009 | 000005.9390 | 00000044325668.0000 | 000154.15 | A TYR 262 A PRO 263 A ARG 264 A ILE 265 A LYS 430 A ASP 431 A GLU 434 A VAL 435

010 | 000005.7930 | 00000056711964.0000 | 000174.63 | A SER 165 A LYS 166 A GLU 196 A HIS 197 A SER 198 A ASP 199 A THR 253 A GLN 256 A THR 257 A PRO 263

011 | 000005.7930 | 00000056711964.0000 | 000126.41 | A TYR 262 A PRO 263 A ARG 264 A ILE 265 A ASP 431 A VAL 435

012 | 000005.6350 | 00000074044064.0000 | 000167.06 | A TYR 262 A PRO 263 A ARG 264 A ILE 265 A LYS 430 A ASP 431 A GLU 434 A VAL 435

013 | 000005.6320 | 00000074419928.0000 | 000161.40 | A TYR 262 A PRO 263 A ARG 264 A ILE 265 A ASP 431 A GLU 434 A VAL 435

014 | 000005.6260 | 00000075177400.0000 | 000145.89 | A TYR 262 A ARG 264 A ILE 265 A LYS 430 A ASP 431 A GLU 434 A VAL 435

015 | 000005.5110 | 00000091281640.0000 | 000193.18 | A ASP 306 A HIS 309 A GLY 310 A THR 382 A ALA 383 A ALA 385 A GLU 386 A ALA 389 A GLU 433 A VAL 437

016 | 000005.5040 | 00000092366504.0000 | 000162.13 | A TYR 262 A PRO 263 A ARG 264 A ILE 265 A LYS 430 A ASP 431 A GLU 434 A VAL 435

017 | 000005.5020 | 00000092678824.0000 | 000162.85 | A GLN 256 A THR 257 A ASN 258 A LEU 259 A VAL 260 A PRO 261 A TYR 262 A MET 313 A ALA 314 A TRP 346 A CYS 347

018 | 000005.2410 | 00000143977968.0000 | 000136.85 | A TYR 262 A PRO 263 A ARG 264 A ILE 265 A ASP 431 A GLU 434 A VAL 435

019 | 000005.2000 | 00000154294144.0000 | 000127.08 | A TYR 262 A PRO 263 A ARG 264 A ILE 265 A ASP 431 A VAL 435

020 | 000005.1800 | 00000159591456.0000 | 000167.29 | A HIS 309 A GLY 310 A LYS 311 A THR 382 A ALA 383 A ALA 385 A GLU 386 A ALA 389 A TYR 432 A GLU 433 A GLY 436 A VAL 437 A ASP 438

021 | 000005.1240 | 00000175411552.0000 | 000154.64 | A HIS 309 A GLY 310 A LYS 311 A THR 382 A ALA 383 A ALA 385 A GLU 386 A GLY 436 A VAL 437 A ASP 438

022 | 000005.1190 | 00000176898128.0000 | 000190.79 | A ASP 306 A HIS 309 A THR 382 A ALA 383 A ALA 385 A GLU 386 A ALA 389 A GLU 429 A TYR 432 A GLU 433 A VAL 437

023 | 000005.1090 | 00000179909184.0000 | 000170.70 | A GLY 310 A THR 382 A ALA 383 A ALA 385 A GLU 386 A ALA 389 A GLU 429 A TYR 432 A GLU 433 A VAL 437

024 | 000004.9670 | 00000228633952.0000 | 000129.35 | A TYR 262 A PRO 263 A ARG 264 A ILE 265 A ASP 431 A VAL 435

025 | 000004.8110 | 00000297502240.0000 | 000161.70 | A GLU 196 A TYR 262 A PRO 263 A ARG 264 A ILE 265 A LYS 430 A ASP 431 A GLU 434 A VAL 435

After clustering the 25 runs, the following 6 distinct complex conformations were found: [They all differ by at least 5.0 A heavy atom RMSD after superposing on the receptor]

Clu |Bind.energy[kcal/mol]|Dissoc. constant [pM]| Con.Surf[A^2] | Contacting receptor residues

----+---------------------+---------------------+---------------+-----------------------------

001 | 000006.8420 | 00000009654894.0000 | 000204.32 | A ASP 306 A HIS 309 A GLY 310 A THR 382 A ALA 383 A ALA 385 A GLU 386 A TRP 388 A ALA 389 A GLU 429 A LYS 430 A TYR 432 A GLU 433 A VAL 437

002 | 000006.3980 | 00000020426964.0000 | 000201.77 | A SER 158 A GLY 162 A LYS 163 A LYS 164 A SER 165 A LYS 166 A GLU 196 A HIS 197 A SER 198 A ASP 199 A GLN 256 A VAL 260 A PRO 263

003 | 000005.9390 | 00000044325668.0000 | 000154.86 | A TYR 262 A PRO 263 A ARG 264 A ILE 265 A LYS 430 A ASP 431 A GLU 434 A VAL 435

004 | 000005.5110 | 00000091281640.0000 | 000190.57 | A ASP 306 A HIS 309 A GLY 310 A THR 382 A ALA 383 A ALA 385 A GLU 386 A ALA 389 A GLU 433 A VAL 437

005 | 000005.5040 | 00000092366504.0000 | 000161.46 | A TYR 262 A PRO 263 A ARG 264 A ILE 265 A LYS 430 A ASP 431 A GLU 434 A VAL 435

006 | 000005.5020 | 00000092678824.0000 | 000161.77 | A GLN 256 A THR 257 A ASN 258 A LEU 259 A VAL 260 A PRO 261 A TYR 262 A MET 313 A ALA 314 A TRP 346 A CYS 347

While the table above lists the best binding energy in each cluster, it is sometimes helpful to also look at the energy spread [average and standard deviation], the dissociation constant has been recalculated from the average binding energy:

Clu |Members|Bind.energy spread [kcal/mol]|Dissoc. constant [pM]

----+-------+-----------------------------+---------------------

001 | 007 | 000006.0987+-000000.6616 | 00000033854000.9050

002 | 004 | 000006.0870+-000000.2223 | 00000034529999.7078

003 | 009 | 000005.4271+-000000.3649 | 00000105171635.0908

004 | 003 | 000005.2480+-000000.1861 | 00000142294444.8352

005 | 001 | 000005.5040+-000000.0000 | 00000092371618.1335

006 | 001 | 000005.5020+-000000.0000 | 00000092684007.8720
